# Diversification of Lipopeptide Analogues Drives Versatility in Biological Activities

**DOI:** 10.1021/acs.jafc.4c11372

**Published:** 2025-01-06

**Authors:** Montserrat Grifé-Ruiz, Jesús Hierrezuelo-León, Antonio de Vicente, Alejandro Pérez-García, Diego Romero

**Affiliations:** Instituto de Hortofruticultura Subtropical y Mediterránea La Mayora, Universidad de Málaga-Consejo Superior de Investigaciones Científicas, Departamento de Microbiología, Universidad de Málaga, Málaga 29071, Spain

**Keywords:** cyclic lipopeptides, structural variants, analogues, *Bacillus velezensis*, antifungal, plant growth promotion, biotechnology, sustainable agriculture, food control.

## Abstract

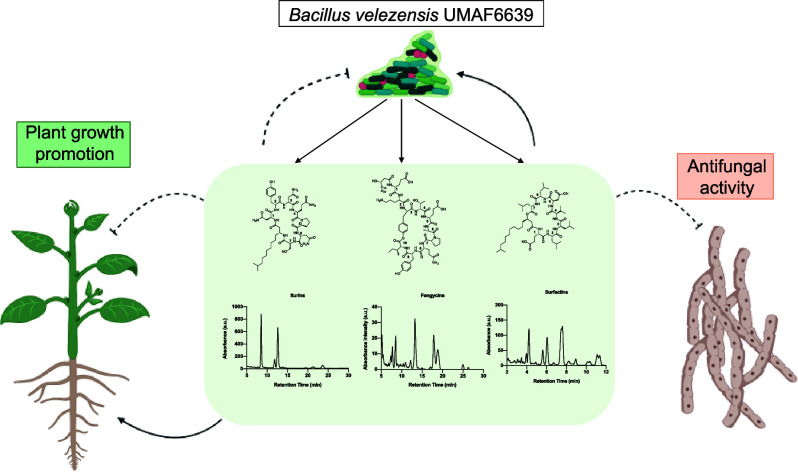

Cyclic lipopeptides (CLPs) are potent secondary metabolites with diverse biological functions. *Bacillus* strains primarily produce CLPs of three key families, namely, iturins, fengycins, and surfactins, each comprising structural variants characterized by a cyclic peptide linked to a fatty acid chain. Despite extensive research on CLPs, the individual roles of these analogues and their proportion in driving biological activity have remained largely overlooked. In this study, we purified and chemically characterized CLP variants from *Bacillus velezensis* UMAF6639 and tested them individually for their antifungal and plant growth-promoting effects. We isolated 5 fractions containing iturin A analogues (from C_13_ to C_17_), 5 fengycin fractions (containing C_16_, C_17_, and C_18_ fengycin A and C_14_, C_15_, C_16_, and C_17_ fengycin B), and 5 surfactin fractions (from C_12_ to C_16_). We show how antifungal activity and seed radicle growth promotion relied on the lipopeptide structural variant and concentration based on the physiological ratio calculated for each lipopeptide variant. Notably, we found that the most toxic variants were the least abundant, which likely minimized autotoxicity while preserving bioactivity. This balance is achieved through synergistic interactions with more abundant, less aggressive analogues. Furthermore, certain fengycin and surfactin variants were shown to increase bacterial population density and exopolysaccharide production, crucial strategies for microbial competition with significant ecological impacts. In addition to advancing basic knowledge, our findings will support the development of precision biotechnological innovations, offering targeted solutions to drive sustainable food production and preservation strategies.

## Introduction

**Figure 1:**
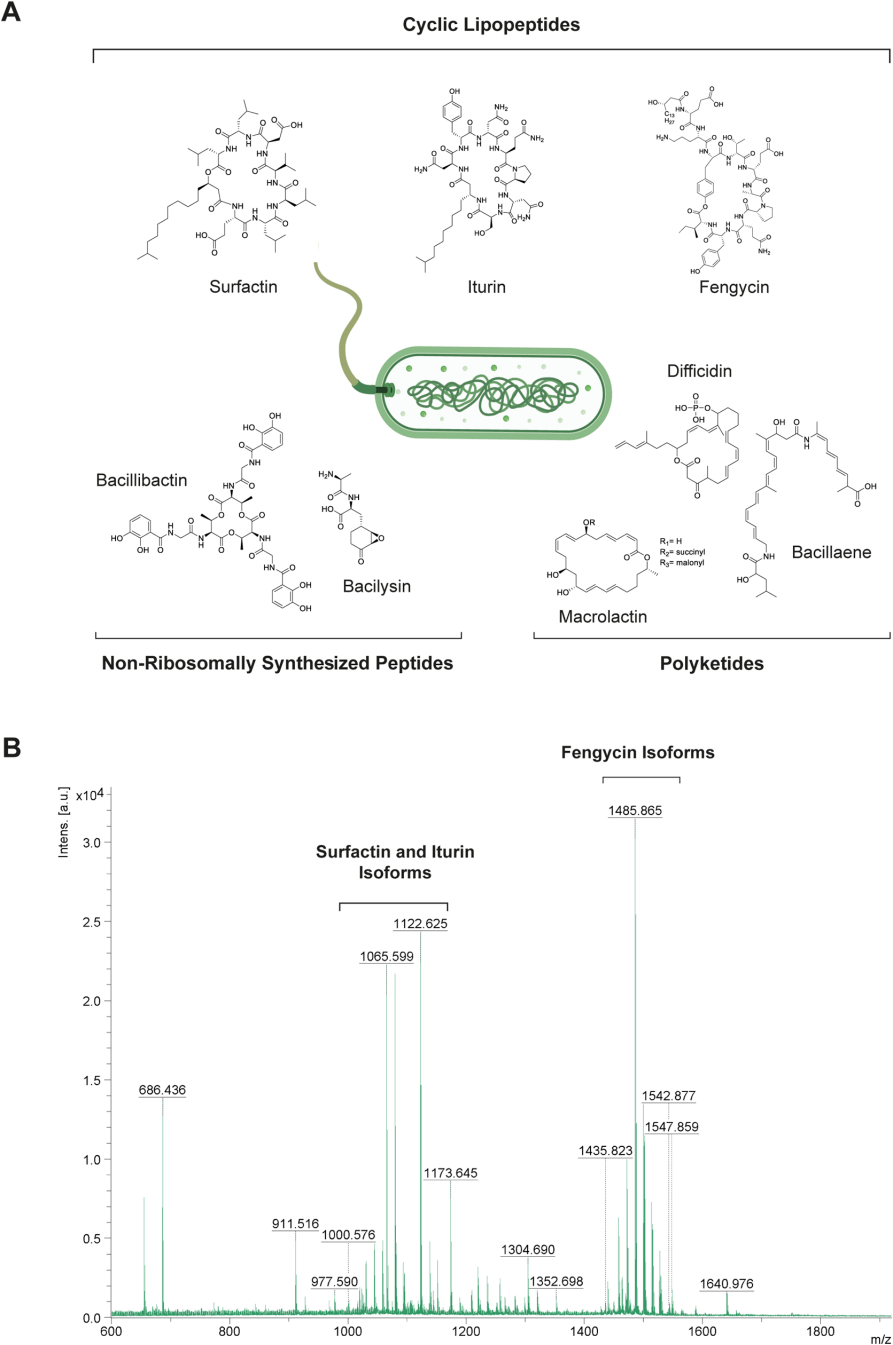
Representative secondary metabolites synthesized by *Bacillus velezensis*. (A) Chemical structure of the most commonly synthesized secondary metabolites. (B) Mass spectra of the three major lipopeptide families found in the *B. velezensis* UMAF6639 supernatant, including iturins, fengycins, and surfactins.

The use of biocontrol agents (BCAs) has been increased substantially in an attempt to mitigate the negative effects of the overuse of chemicals in agriculture. These microorganisms are commonly used as versatile alternatives in the implementation of new techniques aimed at promoting sustainable agriculture.^1^ One of the most representative bacterial BCAs belongs to the *Bacillus velezensis* group, including plant-associated strains known to have common traits related to plant growth promotion, elicitation of the plant immune response and antagonism against phytopathogens.^2,3^ An additional ecological advantage of BCAs is their ability to form biofilms that ensure efficient colonization and persistence in different plant organs.^4^ Moreover, the ability to form endospores is considered a key factor for the efficient formulation of commercial products based on *Bacillus* cells and for prolonged shelf life of the products during storage.^5^ The fact that *Bacillus* strains also produce a battery of secondary metabolites with antimicrobial activities, and likely other unexplored biological functions, is of interest in the agricultural and biotechnological industries.^6^ The wide variety of bioactive compounds is evidenced by the significant amount of genome dedicated to the production of these compounds, reaching up to 10% in *B. velezensis* FZB42, the main representative of the *B. velezensis* group.^5^ In this arsenal of bioactive molecules, antimicrobials such as nonribosomally synthesized peptides (NRPs) and polyketides (PKs) are particularly noteworthy^7^ (Figure 1A). Among the NRPs, cyclic lipopeptides (CLPs) are well characterized molecules in the study of *Bacillus* ecology and the interactions of *Bacillus* species with other organisms.^8^ The three main lipopeptide families found in *B. velezensis* are iturins, fengycins and surfactins. Iturins are heptapeptides linked to a β-amino fatty acid chain with a length of 14–17 carbons and are known to have exceptional antifungal and hemolytic activities, as well as limited antibacterial activity.^9^ Fengycins are lipodecapeptides with an internal lactone ring in the peptidic moiety and a β-hydroxy fatty acid chain (C_14_–C_18_) that can be saturated or unsaturated.^10^ These molecules are specifically active against filamentous fungi^11^ and have been recently shown to play crucial roles in the metabolic reprogramming of melon seeds to promote plant growth and prime plant defenses against foliar phytopathogens.^12^ Finally, the surfactin family contains structural variants, all of which are heptapeptides interlinked with a β-hydroxy fatty acid (C_13_–C_16_) and are known to be powerful biosurfactants with outstanding emulsification and foaming properties and to participate in bacterial biofilm formation and bacterial swarming motility.^13^ While the antimicrobial activity of these lipopeptides has not been studied as deeply as those of other families, antibacterial, antiviral and insecticidal activities have been attributed to them.^10,14,15^ The study and characterization of these molecules requires an understanding of the molecular mechanisms underlying the complex interactions established between the BCAs and the plant, as well as pathogens and other cohabiting microorganisms. Furthermore, the significant cost and technical effort required for the industrial production of these molecules underscore the importance of comprehending their biological activities to direct production efficiently and mitigate common challenges.^16^

Lipopeptides are produced as a mixture of structural analogues, which vary drastically in proportion and composition across *Bacillus* strains.^17^ These changes in the specific composition are also evident between fermentation batches, affecting the consistency of their biological activity. Along with the controversy regarding the mechanisms driving specific biological activities described for some of these lipopeptides, mainly for the surfactin family,^18−20^ this finding supports the idea that distinct analogues may have distinct contributions to the overall biological activities of these molecules. Therefore, this study aimed to explore the specific biological functions of each structural variant of these lipopeptide families, aiming to address the controversial inconsistency in their ecological roles and to ensure robustness in fermentation products to enhance their applicability. To achieve this goal, *B. velezensis* UMAF6639, a biological control agent known for its robust antifungal activity against phytopathogens and as a biofertilizer,^21,22^ was grown in conditions conducive to the production and secretion of lipopeptides. The methods were optimized for efficient separation of the different variants, which were tested for different biological activities. We propose that *Bacillus* produces a specific mixture of lipopeptides as a strategy to avoid the autotoxicity of the most active and less abundant analogues while still preserving their beneficial biological activities. In addition to providing a fundamental understanding of the ecological implications of this bacterial strategy, our findings can be biotechnologically exploited to direct resources toward the production of the most active variants, reducing production costs and, thereby, promoting their implementation in sustainable agricultural practices.

## Materials and Methods

### Microorganisms and Growth Conditions

*B. velezensis* UMAF6639 (CECT8237), which was isolated from the phyllosphere of distinct cucurbit plants,^23^ was obtained from our laboratory strain collection. Bacterial cultures were grown at 28 °C and 150 rpm (when agitation was needed) from frozen stocks in lysogeny broth (LB: 5 g/L NaCl, 5 g/L yeast extract, 10 g/L tryptone, 15 g/L agar). The necrotrophic fungal strain *Botrytis cinerea* Bc05 was obtained from our laboratory strain collection and was grown from frozen stocks on potato dextrose agar (PDA) plates and maintained at 25 °C until sporulation of the culture to perform the corresponding experiments.

### RNA Extraction and RT-qPCR

For RNA extraction, a previously described protocol was followed, albeit with several modifications.^24^ The bacterial strains were cultivated in LB for 24 h, 48 or 72 h at 150 rpm. Biomass was harvested by centrifugation at 12,000 rpm for 5 min and washed twice with 1 mL of PBS. The cells were disrupted by the addition of lysozyme (10 mg/mL) and further incubated at 37 °C for 30 min. After disruption, the mixture was centrifuged for 1 min at 16,000 × g, and the pellets were resuspended in 900 μL of TRI-Reagent (Merck) previously heated at 60 °C. Total RNA extraction was performed according to the manufacturer’s instructions. Genomic DNA removal was carried out using TURBO DNase (Thermo Fisher Scientific) following the manufacturer’s instructions. The integrity and quality of the total RNA were assessed via gel electrophoresis and with a Qubit 3.0 assay. Quantitative real-time (RT–qPCR) was performed via the iCycler-iQ system and the iQ SYBR Green Supermix Kit (Bio-Rad). The primer pairs used to amplify the target genes were designed via Primer3 software (http://bioinfo.ut.ee/primer3/) using previously described parameters.^25^ For the RT–qPCR assays, the RNA concentration was adjusted to 100 ng/μL. Next, 1 μg of DNA-free total RNA was reverse transcribed to cDNA using SuperScript III reverse transcriptase (Invitrogen) and random primers in a final reaction volume of 20 μL according to the instructions provided by the manufacturer. The RT–qPCR cycling conditions were as follows: 95 °C for 3 min, followed by a 40-cycle amplification program (95 °C for 20 s, 60 °C for 30 s, and 72 °C for 30 s) and a third step at 95 °C for 30 s. To normalize the data, the *rpsJ* gene was used as a reference gene.^26^ The target genes and their corresponding primer pairs are listed in Table S1. The relative transcript abundance was estimated via the ΔΔ*c*ycle threshold (Ct) method.^27^ The transcriptional data are presented as the fold changes in the expression levels of the target genes relative to the expression levels at 24 h. RT–qPCR analyses were performed three times (technical replicates) using three independent RNA isolations (biological replicates).

### Lipopeptide Recovery

Iturin, fengycin and surfactin analogues were extracted by acid precipitation of cell-free supernatant (CFS) from *B. velezensis* UMAF6639 cultures by adding 6 N HCl until reaching pH 2, followed by a 24 h incubation at 4 °C to ensure lipopeptide precipitation. The mixture was centrifuged for 20 min at 10,000 rpm to recover the crude lipopeptide extract and incubated in methanol for 5 h. The extract was concentrated in a rotary evaporator at 45 °C and 200 rpm. After evaporation, the crude extracts were reconstituted in 20% acetonitrile (ACN). For the production dynamics analysis, the culture volume was set to 20 mL, and the culture was incubated for 24 h, 48 or 72 h. For preparative purification of the lipopeptide analogues, extraction was performed from 6-L cultures; 24 h cultures were used for fengycin purification, and 48 h cultures were used for iturin and surfactin purification.

### Lipopeptide Analog Purification

The first separation was performed via solid-phase extraction (SPE) on a Strata C18–U column (200 mg, Phenomenex) previously activated with 1 column volume (CV) of methanol. The columns were preconditioned with 1 CV of water, and sample loading was performed with 3 mL aqueous solutions. For lipopeptide elution, the ACN concentration was gradually increased to 100%. Each elution step was monitored by analytical reverse-phase high-performance liquid chromatography (RP-HPLC) analysis. Analog purification was performed via semipreparative RP-HPLC. All the analytical steps were performed with an Eclipse plus C18 5 μm, 4.6 × 250 mm, column (Agilent), and the preparative steps were performed with an Eclipse XDB C18 5 μm, 9.4 × 250 mm column (Agilent). Iturins, fengycins and surfactins were detected via absorbance measurements at 210 nm. The crude extracts obtained from SPE were initially separated to enrich the samples for each lipopeptide family, for which the mobile phase consisted of 0.1% trifluoroacetic acid (TFA) water (A) and ACN (B). An analytical method was developed to determine the retention time (RT) of lipopeptides in the mixture. For this purpose, a gradient was employed with a flow rate of 1 mL/min, with the composition of the mobile phase changing as follows (with time points shown in minutes): T0:100% A, T1:20% A–80% B, T8:60% A–40% B, T25:60% A–40% B, T35:40% A–60% B, T50:40% A–60% B, T60:20% A–80% B, T100:20% A–80% B. For the preparative method, 50 μL samples were injected successively, and similar conditions were utilized, with the flow rate of the mobile phase changed to 2 mL/min and the same gradient applied at the time points 0, 1.5, 11.5, 35.5, 50, 71, 85, and 140 min. To determine the RTs corresponding to each structural variant within every lipopeptide family, the mixtures were subjected to analytical separation under the same aforementioned conditions. Finally, a preparative method was used to purify each analog for further analysis. For this separation, an isocratic method was used for each family. The composition of the mobile phase was 60% A–40% B (30 min) for iturins, 50% A–50% B (30 min) for fengycins and 20% A–80% B (15 min) for surfactins. To determine the physiological concentration of each analog, calibration curves were obtained by applying different concentrations in analytical RP-HPLC under the same conditions as those used with the preparative method, setting the flow rate of the mobile phase to 1 mL/min. After separation, each fraction was evaporated and reconstituted in methanol for further analysis.

### Mass Spectrometry Analysis

The purified lipopeptide analogues were characterized via matrix-assisted laser desorption ionization-time-of-flight (MALDI-TOF) spectrometry. The dried droplet method was used to prepare the samples for MALDI analysis. Briefly, samples were mixed in an Eppendorf tube at a 1:1 ratio with 2,5-dihydroxybenzoic acid (DHB), an α-cyano-4-hydroxycinnamic acid (α-CHCA) matrix or an α-CHCA-DHB matrix mixture (7:3). Each matrix was prepared at a 15 mg/mL concentration and dissolved in TA50 (50% [v/v] ACN and 0.1% [v/v] TFA in distilled water). Then, a 2 μL volume of the sample-matrix mixture was spotted on a stainless steel sample plate and allowed to dry for 10 min at room temperature. The experiments were conducted via an ultrafleXtreme MALDI-TOF MS instrument (Bruker Daltonics) equipped with a 337 nm pulsed nitrogen laser and operated in reflection positive mode with flexControl software (version 3.4; Bruker Daltonics). The laser power was manually adjusted until the optimum signal-to-noise ratio was obtained, and each acquired spectrum resulted from the accumulation of a minimum of 3000 laser shots. MALDI-TOF MS/MS coupled with LIFT mode in the same spectrometer was used to analyze the fragment ions of the selected precursor ions. The spectra were analyzed via Flex Analysis software (Bruker Daltonics).

### Calibration of Lipopeptide Structural Variants

To determine the structural variant ratio produced by *B. velezensis* UMAF6639 (physiological concentration of each analog) and thereby, to know the optimal working concentrations of each lipopeptide variant, a calibration procedure was performed. Purified fractions containing the same variants, as identified by double-fragmentation analysis, were combined. Analytical RP-HPLC was used to generate calibration curves for each analog, where the chromatographic conditions were identical to those previously described for the analytical procedure. Increasing concentrations of each purified lipopeptide variant were injected to construct a standard calibration curve (Figures S8–S10), obtaining the linear regression equations needed for concentration calculation (Table S2). For analogues distributed across multiple peaks in the chromatogram, the total area from all associated peaks was assumed for calibration curve calculations. To calculate the physiological concentrations produced under laboratory conditions of each lipopeptide analog, 5 mL cultures of *B. velezensis* UMAF6639 were incubated under the optimal growth conditions previously specified for each family and were further subjected to acid precipitation and methanol extraction for lipopeptide extraction. After, crude lipopeptide extracts were subjected to analytical RP-HPLC in the same previously described conditions. Using the purified and characterized lipopeptide analogues as standards for fraction identification by RT, the peak area corresponding to each structural variant was used for concentration calculation (Table S3) based on the linear regression equations obtained from the calibration. This experiment was performed by triplicate to ensure accuracy in the concentration calculation.

### Promotion of Radicle Growth Evaluation

Melon seeds (Rochet Panal - Fitó) were surface sterilized with 0.1% sodium hypochlorite and washed twice with distilled water. Seed treatment with the purified variants was performed by bathing the seeds for 1 h at room temperature. The analog concentrations were set to the physiological concentrations obtained from previous calibrations. In addition, a second concentration was assayed as if each analog was the only one produced to reach the same concentration as that of the mixture of each lipopeptide family (Table S3). Since each lipopeptide fraction was reconstituted in methanol, water-treated seeds with the highest concentration of methanol used for lipopeptide treatment were used as control. The methanol concentration ranged from 0.004 to 0.025% for iturins, 0.009 to 0.067% for fengycins and 0.018 to 0.050% for surfactins. The seeds were placed in Petri dishes with moist filter paper and maintained in growth chambers at 25 °C for 5 d under dark conditions. The radicle growth-promoting effects were analyzed on the basis of the radicle areas measured 5 days after seed treatment using Fiji software.^28^

### Antifungal Activity Assay

*B. cinerea* spores were harvested from sporulated plates with distilled water and filtered through a 0.45 μM pore membrane to avoid mycelial contamination. 96-well plates containing 100 μL of PDB with 100 spores per well were used, and the corresponding analog concentrations were analyzed for antifungal activity. H_2_O_2_ (10 mM) was used as a positive control to induce fungal death. For this experiment, methanol concentrations used for lipopeptide treatment ranged from 2 to 6% for iturins, 0.08 to 0.5% for fengycins and 0.2 to 4% for surfactins. The highest methanol concentration used for each condition was added to the control. The plates were incubated for 24 h with agitation (150 rpm) at 25 °C. After incubation, the OD_600_ values of the inoculated plates were measured in a plate reader (FLUOstar Omega reader, BMG LabTech) to evaluate fungal growth within the treatments.

### Assessment of Lipopeptide Analog Bacterial Toxicity

To analyze the toxicity of the lipopeptide variants toward *B. velezensis* UMAF6639, the wells of 96-well plates with 200 μL of LB medium were inoculated with 5 μL of a bacterial suspension (OD_600_ = 1), and every analog was added at the desired concentration.

Methanol concentrations used for lipopeptide treatment in each condition ranged from 0.005 to 4.3%, and the maximum was used as a control to confirm that was not harmful for *Bacillus* growth. OD_600_ values were measured in a plate reader (FLUOstar Omega reader, BMG LabTech) in kinetic mode with temperature control (28 °C), with measurements taken every 20 min.

### Evaluation of Extracellular Matrix Component Production

To evaluate the effects of separate structural variants on the expression of the main extracellular matrix (ECM) components, two separate *Bacillus subtilis* NCIB3610 strains expressing transcriptional fusions of the main promoter of the exopolysaccharide (EPS) operon or the structural protein TasA to fluorescent proteins were used for confocal laser scanning microscopy (CLSM) analysis. Analog treatments were carried out as previously described for the toxicity assays. Twenty microliters of each bacterial culture were taken at 14 h after CLP treatment, corresponding to the highest OD_600_ values detected, and placed onto 1% agarose-covered slides. CLSM image acquisition was performed with excitation at 488 nm and emission recorded from 520 to 620 nm. All images were obtained by visualizing the samples using an inverted Leica SP5 system with a 63x NA 1.4 HCX PL APO oil-immersion objective. For each experiment, the laser settings, scan speed, PMT or HyD detector gain, and pinhole aperture were kept constant for all of the acquired images. Image processing was performed via Fiji software,^28^ where several individual fluorescence intensity measurements for each condition were recorded for mean fluorescence intensity quantification. Bacterial counts of the same cultures were carried out by performing serial dilutions and spreading 100 μL of the cell suspension onto LB plates that were further incubated at 28 °C for 24 h.

## Results

### Lipopeptide Analogues Are Differentially Produced in *B. velezensis* UMAF6639

**Figure 2:**
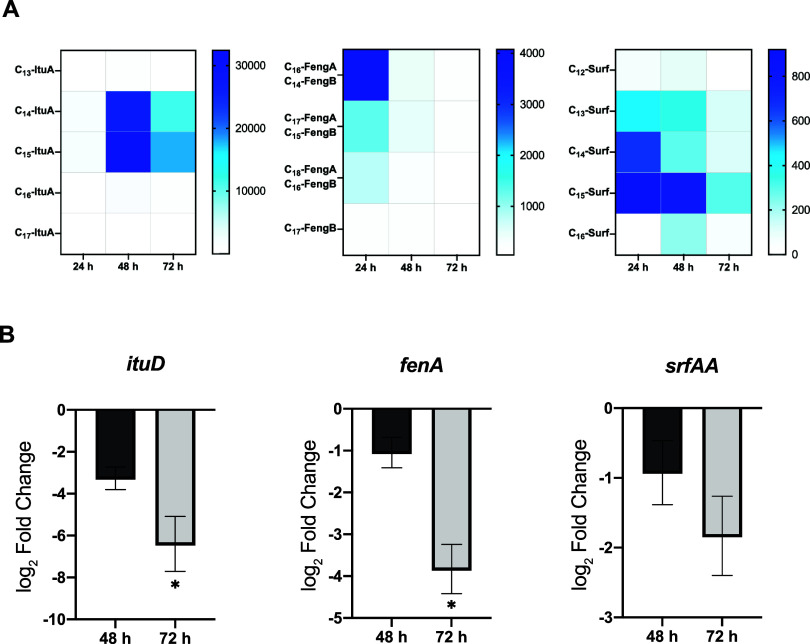
Production dynamics of cyclic lipopeptides in *B. velezensis* UMAF6639. (A) Accumulation dynamics of the lipopeptide analogues. Heatmaps illustrating the mean peak intensity obtained via MALDI-TOF mass spectrometry analysis, where color intensity reflects the relative abundance of each structural variant across time. (B) Expression pattern of the first gene of each biosynthetic operon (*ituD* for iturin, *fenA* for fengycin. and *srfAA* for surfactin biosynthesis), normalized to expression levels at 24 h.

To determine the optimal fermentation time that ensured the greatest accumulation of lipopeptides, 20 mL cultures of *B. velezensis* were incubated at 28 °C, and samples were taken at 24 h, 48 and 72 h for further analysis. After organic extraction of the spent medium, the whole analog profile of each lipopeptide family was obtained via MALDI-TOF analysis. Traces within a mass range from *m*/*z* 990–1130, corresponding to iturins and surfactins, and those within a mass range from *m*/*z* 1460–1550, associated with fengycins, were detected (Figure 1B). To estimate the lipopeptide production and accumulation dynamics, each structural variant was considered separately, and the mean peak intensities corresponding to the previously described *m*/*z* values for characterized lipopeptide es, including their corresponding Na^+^ and K^+^ adducts, were measured (Figure 2A). C_14_-iturin A and C_15_-iturin A were predominantly detected, and their accumulation levels peaked at 48 h. The concentrations of C_16_-fengycin A/C_14_-fengycin B, the most abundant analogues of fengycin, peaked at 24 h. C_13_ to C_15_-surfactin represented the majority of variants within the surfactin family, and although the maximum accumulation was recorded at 24 h, the optimal production time was established at 48 h, given that the C_12_ and C_16_ analogues were not detected at significant levels at 24 h. According to this chemical analysis, RT–qPCR revealed repression of the expression of the first gene of each biosynthetic operon at 48 h (Figure 2B) and the greatest repression after 72 h of growth.

### Identification and Characterization of Lipopeptide Analogues via RP-HPLC and MALDI-TOF Spectrometry

**Figure 3:**
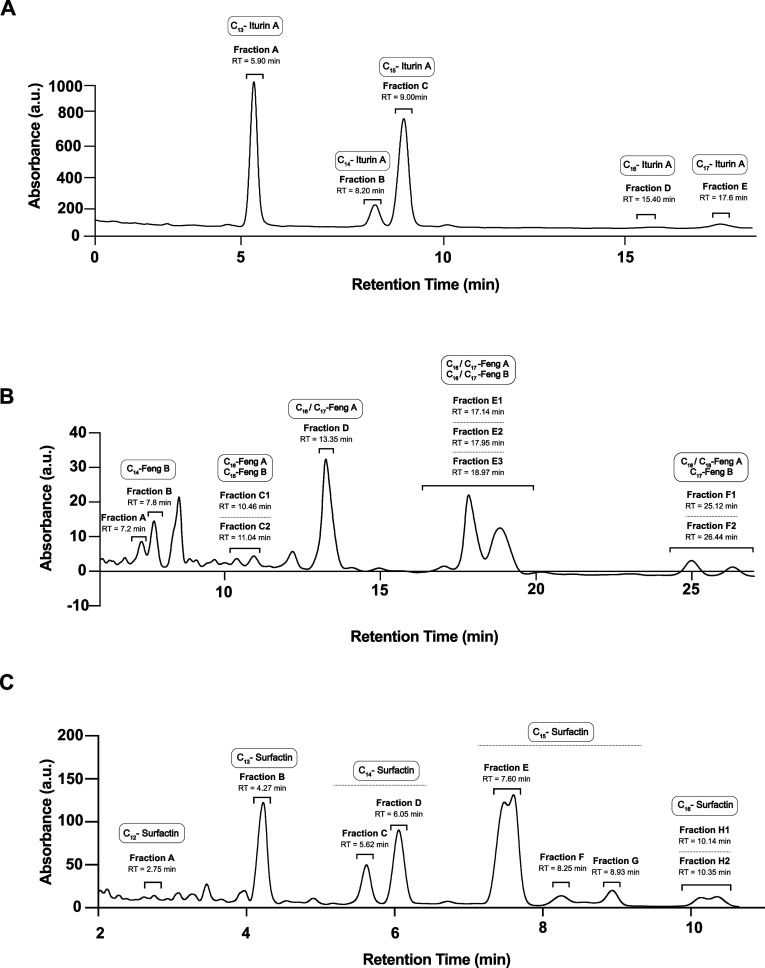
Analytical HPLC chromatograms representing the three major cyclic lipopeptide families found in *B. velezensis* UMAF6639 after SPE separation. Peak profiles corresponding to (A) iturins, (B) fengycins, and (C) surfactins. The upper labels represent the retention time (RT) for each peak, grouped peaks in the same fraction, and the corresponding lipopeptide analogue detected by mass spectrometry.

Once the fermentation time was specifically defined for the production of each molecular family, the lipopeptides were harvested via acid precipitation, methanol extraction and rotary evaporation. The crude extracts were fractionated via SPE chromatography for the initial separation of the three families, which facilitated further separation and increased the purity for RP-HPLC analysis (Figure S1). Initial analytical separation revealed a complex mixture of analogues within each molecular family. These variants were fractionated under semipreparative conditions to achieve purification prior to further analysis. The separation of iturins and fengycins yielded five fractions (Figure 3A,B). However, fractions C, E, and F of fengycins were integrated in two consecutive peaks that were not further separated due to technical limitations. Finally, eight fractions were recovered from surfactins Figure 3C), with fraction H also including two consecutive peaks.

Fractions collected via semipreparative RP–HPLC were analyzed MALDI–TOF mass spectrometry to characterize the lipopeptide analogues. Some of the structural variants exhibited the same *m*/*z* values from MS analysis; thus, a double fragmentation analysis was performed to determine the identity of their structure and the amino acid sequence of the peptide moiety. For the iturin family, the fragmentation patterns of the parent ions from fractions A to E with *m*/*z* 1029, 1043, 1057, 1093, and 1107, respectively, demonstrated differences in the length of the fatty acid chain and permitted the identification of the C_13_ to C_17_ analogues (Figure S2). All iturin analog fragments corresponded to the previously described iturin A,^29^ with the peptide sequence Ser-Asn-Pro-Gln-Asn-Tyr-Asn (Figure S3). Fengycin fraction A was excluded from further analysis given that the MS spectrum revealed contamination with C_17_-iturin A. Within the remaining fractions of this molecular family, precursor ions with *m*/*z* 1464, 1478, 1492, and 1506 exhibited different double fragmentation patterns for each fraction (Figure S4). These peaks corresponded to putative fengycins A and B, with the amino acid sequence Glu-Orn-Tyr-Thr-Glu-Ala/Val-Pro-Gln-Thr-Ile. Changes in the amino acid at position 6 have been shown to discriminate between two variants, namely, fengycin A (Ala) and fengycin B (Val), as evidenced by fingerprint peaks in the spectra (*m*/*z* 1080 and 966 for fengycin A; 1108 and 994 for fengycin B).^30^ On the basis of these fragmentation patterns, seven fengycin variants were identified with changes in the amino acid and fatty acid moieties: C_16_, C_17_ and C_18_ for fengycin A and C_14_, C_15_, C_16_ and C_17_ for fengycin B (Figure S4). Five surfactins were identified by MS/MS of the precursor ions with *m*/*z* 995, 1009, 1023, 1051, 1059, and 1075 (Figure S6). Variations in the *m*/*z* values of the resulting fragments were correlated with changes in the length of the lipidic part of the molecule, retaining the same amino acid sequence: Glu-Leu/Ile-Leu-Val-Asp-Leu-Leu/Ile.^31^ Based on these observations, surfactins from C_12_ to C_16_ were identified (Figure S7). Following analog characterization, the physiological concentration of each lipopeptide family, along with each structural variant separately, was calculated to perform further biological experiments (Tables S2 and S3).

### Biological Activity of Lipopeptide Analogues Is Dependent on the Structure and Concentration

The purified and chemically characterized analogues were evaluated for antifungal activity against *B. cinerea* and melon seed radicle growth promotion activities, two biological activities previously shown to be associated with these lipopeptides.^12,32−34^ The different structural variants were tested either alone or in combination at two different concentrations: they were tested (i) at the level physiologically produced by bacteria under laboratory conditions and (ii) by increasing the concentration of each analog to reach the level of the analog mixture (Table S3).

**Figure 4:**
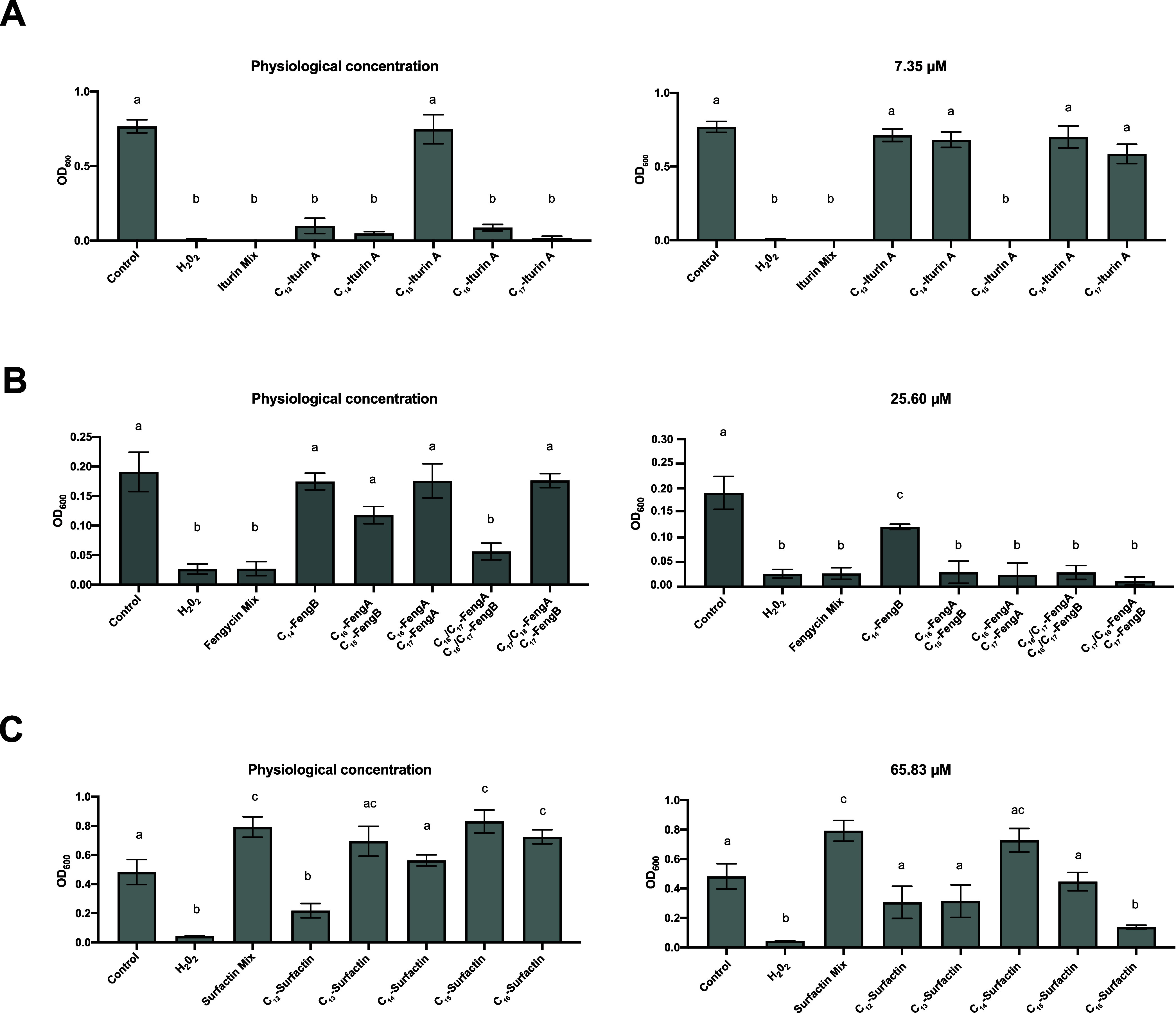
CLP analogues show strong differences in antifungal activity when applied separately. The OD_600_ was used as an indicator of fungal growth in the presence of (A) iturin, (B) fengycin, and (C) surfactin analogues. Fungal growth was measured at the physiological concentration of each analogue and at the mixture concentration. The error bars represent the standard error of the mean (SEM), and one-way ANOVA was used to analyze the statistical significance of the results (*p* < 0.05). The letters above the columns represent the group conditions without statistically significant differences.

The antifungal activity assays revealed complete inhibition of fungal growth in the presence of the iturin A mixture, an expected finding based on previous studies.^23,35^ When tested separately at their physiological concentrations, only C_15_-iturin A failed to exhibit antifungal activity (Figure 4A, left). However, the administration of each structural variant at the concentration of the entire mixture (7.35 μM) resulted in C_15_-iturin A being the sole active analog (Figure 4A, right). The fraction containing C_16_/C_17_-fengycin A and C_16_/C_17_-fengycin B was statistically grouped only with the positive control and with the fengycin mixture (Figure 4B, left), which reduced fungal growth. For the highest concentration tested, all the variants showed excellent antifungal properties, except C_14_-fengycin B, which retained only weak antifungal activity that was not comparable to the effects of the other molecules (Figure 4B, right). The surfactin mixture and the purified analogues showed no significant antifungal activity at physiological concentrations, except for the C_12_ analog. In fact, an increase in fungal growth was observed for most of the fractions, including the mixture (Figure 4C, left). When the concentration increased, fungal growth decreased, but only the antifungal activity of the C_16_ variant was comparable to that of the positive control (Figure 4C, right).

**Figure 5:**
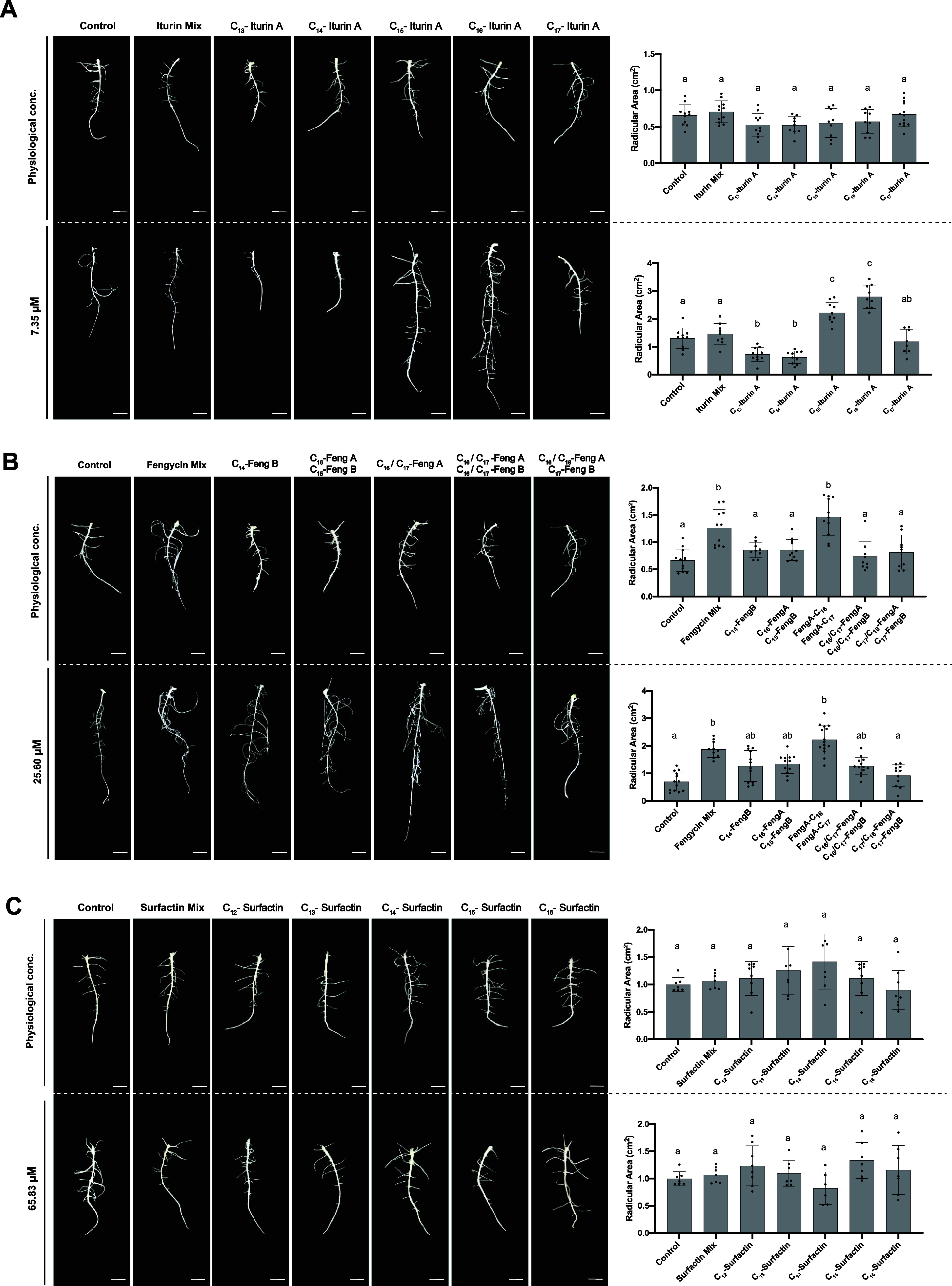
Chemical structure and concentration impact the plant growth promotion potential of CLP analogues used to treat melon seeds. Representative image of the radicle development of melon seedlings 5 days after treatment with (A) iturins, (B) fengycins, and (C) surfactins and the corresponding quantitative measurements of root areas. Radicle area measurements of melon seedlings 5 days after treatment with separate structural variants. The error bars represent the SEM, and one-way ANOVA was used to analyze the statistical significance of the results (*p* < 0.05). The letters above the columns represent the group conditions without statistically significant differences.

To evaluate the potential of each analog in promoting melon seed radicle growth, the radicular development of 5-day-old seedlings was measured. Treatment with the iturin A mixture from the *B. velezensis* UMAF6639 supernatant did not promote radicle growth. Similarly, no promotion of radicle growth was recorded when seeds were treated with the analogues separately at their physiological concentrations (Figure 5A, top). At the same concentration, C_13_ and C_14_-iturin A compromised root development, C_17_-iturin A slightly reduced the root area, and the C_15_ and C_16_ variants significantly promoted radicle growth (Figure 5A, bottom). Fengycin treatments resulted in a significant increase in the root area. The fraction containing C_16_ and C_17_-fengycin A showed the same growth promotion potential as the mixture at lower concentrations (Figure 5B, top). Treatment with all the fractions alone at a higher concentration (25.60 μM) promoted root development, with the fractions containing C_16_ and C_17_-fengycin A being the most active (Figure 5B bottom). However, C_17_ and C_18_-fengycin A in combination with C_17_-fengycin B appeared to be ineffective at promoting radicle growth at any of the concentrations tested (Figure 5B). Radicle growth promotion in surfactin-treated melon seeds was not detected for any analog or for the mixture at any concentration assayed (Figure 5C).

### Proportion of Each Analog in the Mixture Determines Autotoxicity in the Producer Strain

**Figure 6:**
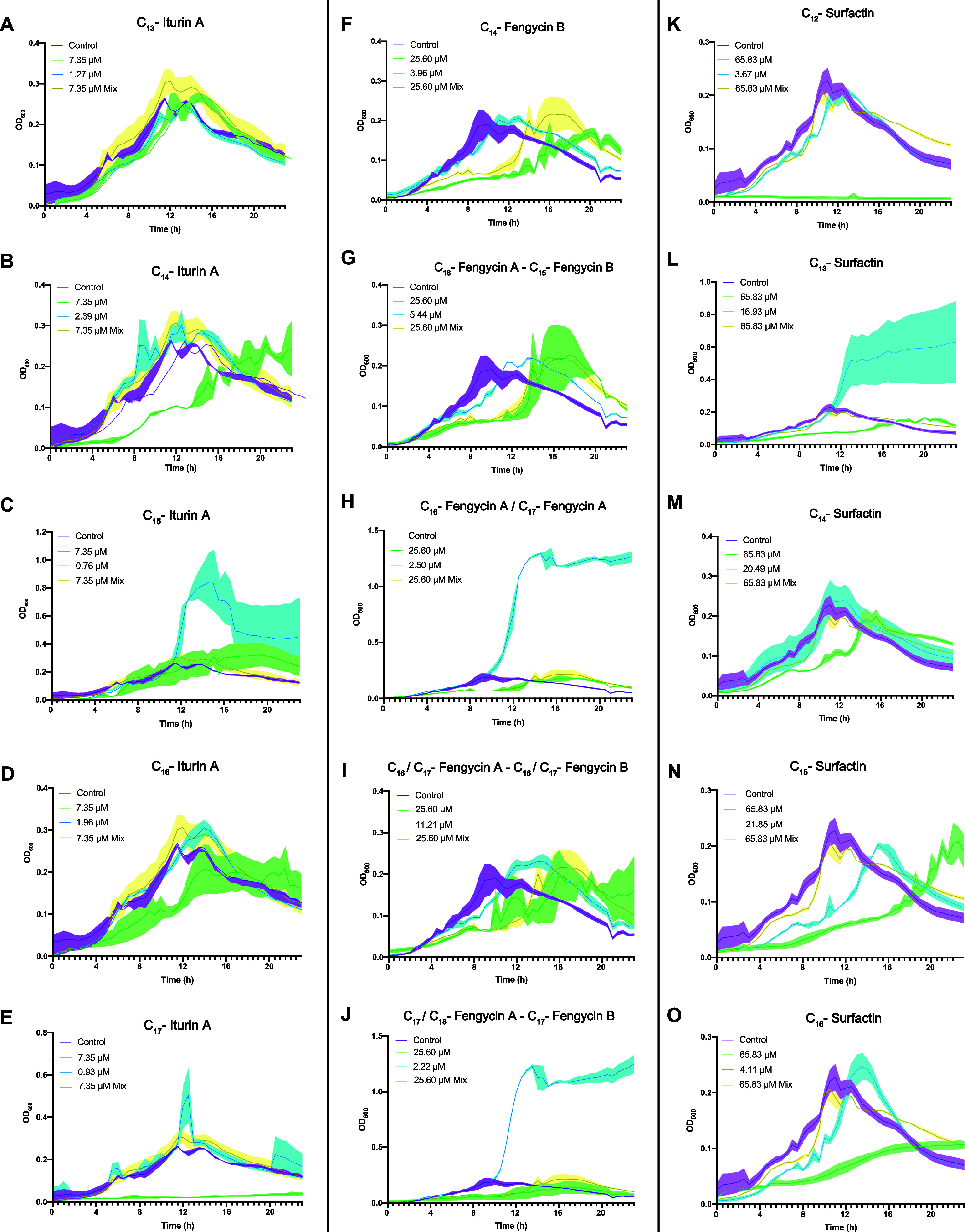
Effect of naturally produced lipopeptide analogues on *B. velezensis* UMAF6639 during cultivation in LB medium. Iturins (A–E), fengycins (F–J), and surfactins (K–O) were tested for their effects on *Bacillus* growth. Cell growth was monitored every 20 min for 24 h. In each experiment, the maximum concentration of methanol used for the treatment with analogues was used as a control (purple), and analogues were tested separately at the concentration of the whole mixture (green) and at the physiological concentrations (blue) and all together at the same proportion as that produced by the bacteria (yellow). Measurement at each time point was performed in triplicate. The graphs represent the mean values, and the error bars (shadows over the lines) represent the SEM.

The fact that some of the most active variants were less abundant led us to speculate on the putative autotoxicity of those molecules at concentrations higher than the physiological concentrations. *B. velezensis* UMAF6639 cultures were treated with each molecule at the aforementioned concentrations, and *Bacillus* growth dynamics were estimated by monitoring the OD_600_ over 24 h (Figure 6). Treatment with the purified iturin A mixture did not significantly affect bacterial growth. C_13_-iturin A was harmless to *Bacillus* cells (Figure 6A). Cultures treated with the C_14_ and C_16_ variants at physiological concentrations showed no difference from the control. However, when the concentration was increased, bacterial growth was slightly delayed (Figure 6B,D). A considerable increase in the OD_600_ value occurred in the presence of C_15_-iturin A at its physiological concentration, and while higher concentrations led to a decrease in the optical density, the values remained above those of the untreated control (Figure 6C). Interestingly, the C_17_ analog at the higher concentration completely arrested *B. velezensis* growth (Figure 6E).

The fengycin mixture delayed bacterial growth, which finally reached the same values as those of the untreated cultures after a lag phase of 8 h. C_14_-fengycin B (Figure 6F), along with the fraction composed of C_16_-fengycin A and C_15_-fengycin B (Figure 6G), slightly delayed growth at physiological concentrations, and this effect was more pronounced at the highest concentration evaluated. The same effect was observed for the fraction containing C_16_/C_17_-fengycin A along with C_16_/C_17_-fengycin B (Figure 6I). Finally, fractions containing C_16_/C_17_-fengycin A (Figure 6H), as well as C_17_/C_18_-fengycin A and C_17_-fengycin B (Figure 6J) at lower concentrations, caused a noticeable increase in the OD_600_ values and retained the same effect as reported for the aforementioned mixture concentration. The surfactin mixture had no effect on *Bacillus* growth according to the optical density measurements. All surfactin structural variants were toxic when applied at the mixture concentration, an expected finding given the strong surfactant characteristics of these molecules, revealing the robust balance needed for bacterial cells to preserve a normal growth rate in the presence of surfactins. Although this effect was observed for all the analogues, the C_12_ (Figure 6K) and C_16_ (Figure 6O) analogues appeared to be the most toxic for UMAF6639, and not surprisingly, this was correlated with the low abundances of these molecules produced by this strain (Table S2). In terms of the effect of the physiological concentration, C_13_-surfactin caused a considerable increase in the OD values (Figure 6L), whereas C_15_ and C_16_ delayed bacterial growth (Figure 6N,O).

**Figure 7:**
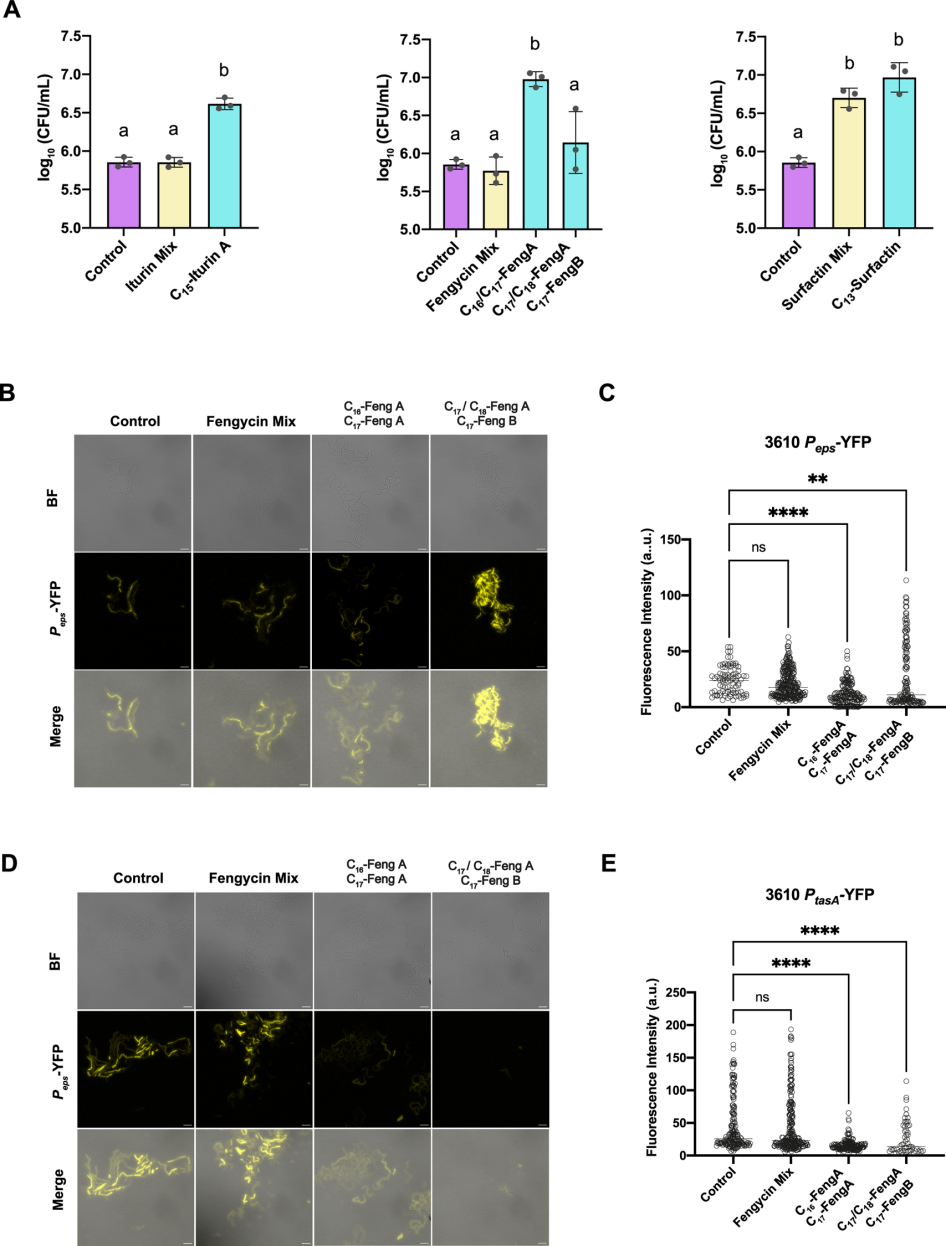
Increase in the OD_600_ values of *Bacillus* cultures treated with different lipopeptide analogues corresponds to an increase in the bacterial count or increased extracellular matrix production, depending on the analogue. (A) Log_10_ (CFU/mL) corresponding to *Bacillus velezensis* UMAF6639 cultures treated with analogues causing an increase in OD_600_ values in the toxicity assay. The highest methanol concentration was used as the control condition (purple), each structural variant mixture was used at the physiological concentration calculated from the previous calibration (yellow, see Tables S2 and S3), and each analogue was used separately at its physiological concentration (blue). The error bars represent the SEM, and one-way ANOVA was used to analyze the statistical significance of the results (*p* < 0.05). The letters above the columns represent the group conditions without statistically significant differences. (B) Impact of fengycin structural variants on *Bacillus* exopolysaccharide production. The reporter strain *Bacillus subtilis* NCIB3610 carrying the transcriptional fusion *Peps-*YFP was used to monitor extracellular matrix production in response to the C_16_/C_17_–FengA and C_16_/C_18_–Feng A-C_17_–Feng B treatments. Scale bars correspond to 10 μm. (C) Fluorescence intensity measurements corresponding to *eps* expression. Each dot represents an individual measurement, and horizontal lines indicate the mean fluorescence intensity with the SEM. Statistical significance was determined via the Kruskal–Wallis test, followed by Dunn’s multiple comparison test. Nonsignificant (ns), ** *p* < 0.01, and **** *p* < 0.0001. (D) Monitoring of the *tasA* expression, encoding the major extracellular matrix protein TasA in the *B. subtilis* strain *P*_*tasA*-_YFP under different fengycin analogue treatments. Scale bars correspond to 10 μm. (E) Fluorescence intensity measurements corresponding to *tasA* expression. Data analysis and representation were performed with the same parameters as those used for the *eps* assay.

To confirm that the increase in the optical density of the cultures could be attributed to a relatively high growth rate, the population size was estimated by CFU counts under each condition when the OD_600_ values exceeded those of the untreated controls (C_15_-iturin A, C_16_/C_17_-fengycin A, C_17_/C_18_-fengycin A and C_17_-fengycin B, and C_13_-surfactin) along with those observed with the mixtures. The increase in absorbance for all the structural variants tested was directly correlated with increased bacterial counts, except for the fraction containing C_17_/C_18_-fengycin A and C_17_ fengycin B (Figure 7A). A putative explanation for this finding might be the overexpression of ECM components. Two *B. subtilis* NCIB3610 reporter strains were used to monitor the expression of key elements for ECM production: *P*_*eps*_*-*YFP, for *epsA-O* exopolysaccharide operon expression, and *P*_*tasA*_*-*YFP, for the expression of the gene encoding the extracellular matrix structural protein TasA. Fluorescence intensity measurements for the CLSM images of these transcriptional fusions revealed that only the fengycin fraction consisting of C_17_/C_18_-fengycin A and C_17_-fengycin B increased the expression of the *eps* operon (Figure 7B,C) and that *tasA* expression was repressed by both fractions of fengycin (Figure 7D,E).

## Discussion

The direct antagonism of CLPs against fungal phytopathogens has been reported to rely on the ability of CLPs to directly interact with biological membranes, which is dependent on their fine composition,^36^ specifically the length of their fatty acid chain. Therefore, it could be assumed that for all the CLP molecular families, the greatest antifungal activity would be displayed by the longest analogues. Despite the well-documented antifungal potential of iturins,^22,37,38^ we have observed significant disparities between the variants and their concentrations. The presence of structural isomers of these molecules^39^ (Table S4), indicates that variations in radical configurations may influence the interaction between iturin A analogues and fungal membranes. Notably, the C_15_ analog, with three possible structural isomers, showed the greatest differences in antifungal activity depending on the assayed concentration. These findings suggest that the nature of the structural isomers and their proportion in the mixture, in addition to the acyl chain length, could play a crucial role in the antifungal activity of iturins. For fengycins, the dose-independent “all-or-none” antifungal activity^40^ explains the lack of effect observed at lower concentrations for most structural variants. As concentrations increase, antifungal effects emerge, suggesting that at physiological levels, most of the analogues may not reach their minimal inhibitory concentration individually but could act synergistically to combat the pathogen.

The biocontrol activity of surfactins has been described primarily as an indirect mechanism, driven by the elicitation of the plant immune system rather than by direct antagonism of the pathogen.^21,41,42^ Some studies have demonstrated how different structural variants of this lipopeptide are able to trigger different immune responses in the plant,^43−45^ but research on how the naturally produced variant ratio influences the biological outcomes still remains scarce. Recent studies with artificial membranes have shown that surfactins interact with biological membranes by causing lipid packing and graded leakage, but they lack the efficient disruptive capacity of fengycins.^32^ These findings align with our results, wherein most of the surfactin variants did not show any significant direct antagonistic activity at physiological concentrations against *B. cinerea*. However, the strong surfactant capacity of surfactins, especially that of the longest analog, indicates that beyond a certain concentration, their membrane disruption effect and antifungal activity could be enhanced.

In another recent study, plipastatins, fengycin analogues produced by *B. subtilis*, were reported to interact with oil bodies (OBs) of the melon seed endosperm, accelerating lipid mobilization and resulting in plant growth promotion (PGP) and immunization.^12^ Treatment with the isolated fengycin variants resulted in different radicle growth promotion phenotypes, and fengycin A (C_16_ and C_17_ analogues) was the most active. Because fengycin B variants have the same fatty acid length, the amino acid at position 6 of the peptide moiety, which differs between fengycin A and B,^46^ is important for their specific biological activities. The specificity of the interaction of these fengycin variants with other organisms is further supported by the variation in the minimal inhibitory concentration of fengycin A, which was notably greater than that of fengycin B, for antagonizing *Fusarium oxysporum* f. sp. *radicis-lycopersici*.^47^ Despite the surfactant properties of surfactins, the differences between surfactins and fengycins in their mode of action on biological membranes^32^ are associated with the absence of PGP activity, given the hypothesized interaction of CLPs with seed OBs. In addition, our findings indicate that only the C_15_ and C_16_ iturin A variants, which uniquely exhibit structural isomers within the iturin A mixture, are able to promote radicle development when applied separately. All iturins share the same amino acid sequence and differ only in lipid chain length; however, an expected increase in activity with longer analogues such as C_17_ was not observed, suggesting that the presence of the aforementioned structural isomers may also determine their PGP activity. The differences observed in the contributions of different CLP analogues to biological activities led us to consider the ecological relevance of the structural variant proportion that is physiologically produced. The fact that different analogues were toxic to the producer strain when their concentrations were increased highlights the ability of their physiological concentrations to maintain a cellular balance.

Ecologically, CLPs are known as part of the mechanisms used to manage competitors in crowded environments such as the rhizosphere but also to support key developmental traits such as cell motility, biofilm formation, and host colonization.^48−50^ We observed that some structural variants increased the optical density of the producer strain culture, which corresponded with either an increase in bacterial counts or overproduction of the extracellular polymer EPS. EPSs have been widely described as not only playing an important role in the aforementioned processes^51^ but also contributing to protecting cells against osmotic and oxidative stress or cryoprotection,^52−55^ all of which provide an ecological advantage in interactions with other microorganisms. Thus, the increased EPS production induced by C_17_-fengycin A and C_18_-fengycin might be hypothesized to modulate cross-species interactions and niche colonization. Our discovery that specific fengycin variants can induce the synthesis of EPS but not TasA (which are the two main components of the *Bacillus* ECM) suggests the presence of a tightly regulated mechanism separated at some point in the biofilm-related regulatory pathways triggered by surfactins and bacillomycin D (a secondary metabolite of the iturin family).^56,57^

Surfactins also play an important role in the interaction of *Bacillus* with other microorganisms.^58,59^ Recent studies have demonstrated how increased surfactin production is an adaptative mechanism forced by the continuous interaction with the pathogen *Aspergillus niger*.^20^ Moreover, Lozano-Andrade et al. demonstrated that surfactin production by *B. subtilis* is crucial for its establishment within a synthetic bacterial community, given that the *srfAC* mutant defective in surfactin production was unable to sustain population levels comparable to those of the wild-type strain.^60^ This observation correlates with the results of the metabolomic analysis performed by Molina-Santiago et al.^61^ on the interaction between *Bacillus* and *Pseudomonas*. This study revealed that the production of the whole surfactin pool increased throughout the interaction, with the C_13_ analog being the most abundant. These findings align with our results on the ability of C_13_-surfactin to increase bacterial population size when applied individually, which supports the ecological relevance of this analog as part of the *Bacillus* arsenal deployed against different pathogens or as a defensive mechanism to survive in interactions with other microorganisms. Our results not only support the previously characterized dynamics of surfactin production by *Bacillus* strains in interaction with other microorganisms but also further demonstrate that population growth in *B. velezensis* is driven predominantly by a single surfactin analog, underscoring the functional diversity within the CLP family and therefore highlighting the ability of distinct structural variants to mediate specific biological functions, including phytopathogen suppression, plant health promotion, and the modulation of intricate microbial interactions within the ecosystem. Our data underscore that the biological activities of CLPs are significantly influenced by both the relative abundance and specific application of individual analogues, with concentration-dependent effects playing a critical role. These observations suggest that future strategies for optimizing CLP production should prioritize the precise modulation of analog composition rather than simply maximizing total yield to achieve targeted biological effects. By elucidating the individual actions of each analog, we provide a valuable framework for designing CLP production and biotechnological product formulation strategies, enabling adjustments to the mixture for specific outcomes on the basis of the intended application. Understanding these dynamics will also help elucidate the delicate balance required for the ecological performance of CLPs, emphasizing the importance of further investigation in this field, which is crucial for improving their biotechnological potential in enhancing sustainable agricultural practices.

## Supporting Information Available

Chromatograms corresponding to SPE purification of the three CLP families; MALDI-TOF mass spectra of each fraction corresponding to the double fragmentation pattern of the three lipopeptide families and the proposed analog amino acid sequence; RP-HPLC chromatograms of lipopeptide variant calibrations for physiological concentration calculations; primers used to quantify the expression levels of lipopeptide genes by RT-qPCR; linear regression equations corresponding to the calibration of each lipopeptide analogue; calculated physiological concentrations of the different lipopeptide structural variants produced by *B. velezensis* UMAF6639; and structure of the fatty acid side chains corresponding to iturin A analogues (PDF)
Complete record of the peak intensities recorded from double fragmentation in mass spectrometry characterization of all lipopeptide structural analogues corresponding to the spectra in Figures S2, S4, and S6 (XLSX)
