# Supplementary material for: Diversification of Lipopeptide Analogues Drives Versatility in Biological Activities
Source: J Agric Food Chem. 2025 Jan 6;73(2):1403–16. doi: 10.1021/acs.jafc.4c11372 (PMC11741111; doi:10.1021/acs.jafc.4c11372)
Supplement: Supplementary file 1 — jf4c11372_si_001.pdf [file jf4c11372_si_001.pdf]

Supporting information

Diversification of Lipopeptide Analogues Drives Versatility in Biological Activities

Montserrat Grifé-Ruiz[1], Jesús Hierrezuelo-León[1], Antonio de Vicente[1], Alejandro Pérez-García[1], Diego Romero[1*]

[1]Instituto de Hortofruticultura Subtropical y Mediterránea La Mayora, Universidad de Málaga-Consejo Superior de Investigaciones Científicas, Departamento de Microbiología, Universidad de Málaga, Málaga, Spain

*** Corresponding author:**
diego_romero@uma.es

Supplementary figures

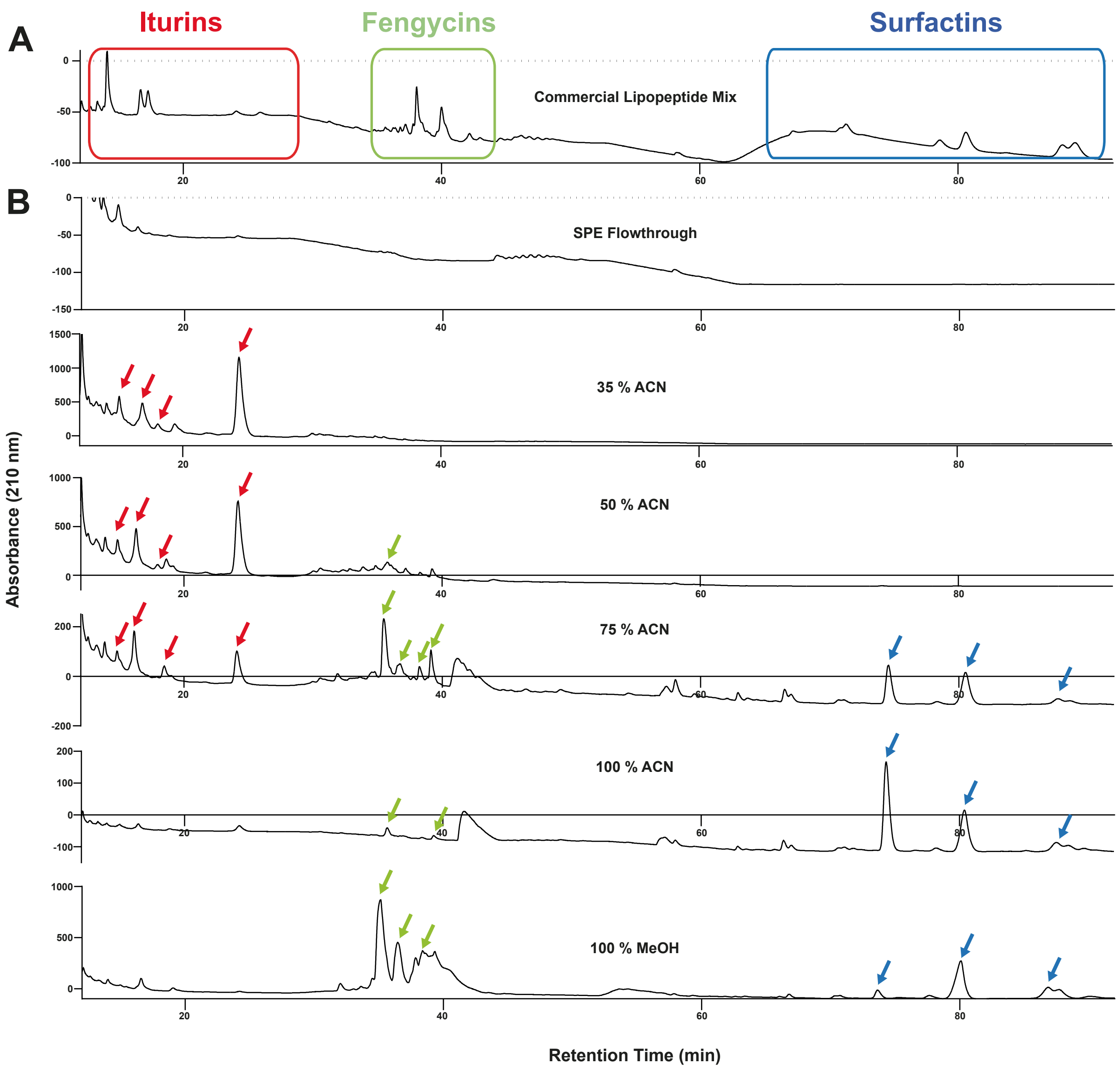

Figure S1. Cyclic lipopeptide analog purification. (A) Analytical HPLC chromatogram representing peaks corresponding to the three major lipopeptide families using a commercial mix as standard for RT identification of each family. **(B)** Analytical HPLC chromatograms corresponding to different acetonitrile gradients for lipopeptide elution in SPE chromatography. iturins: red arrows, fengycins: green arrows, surfactins: blue arrows.

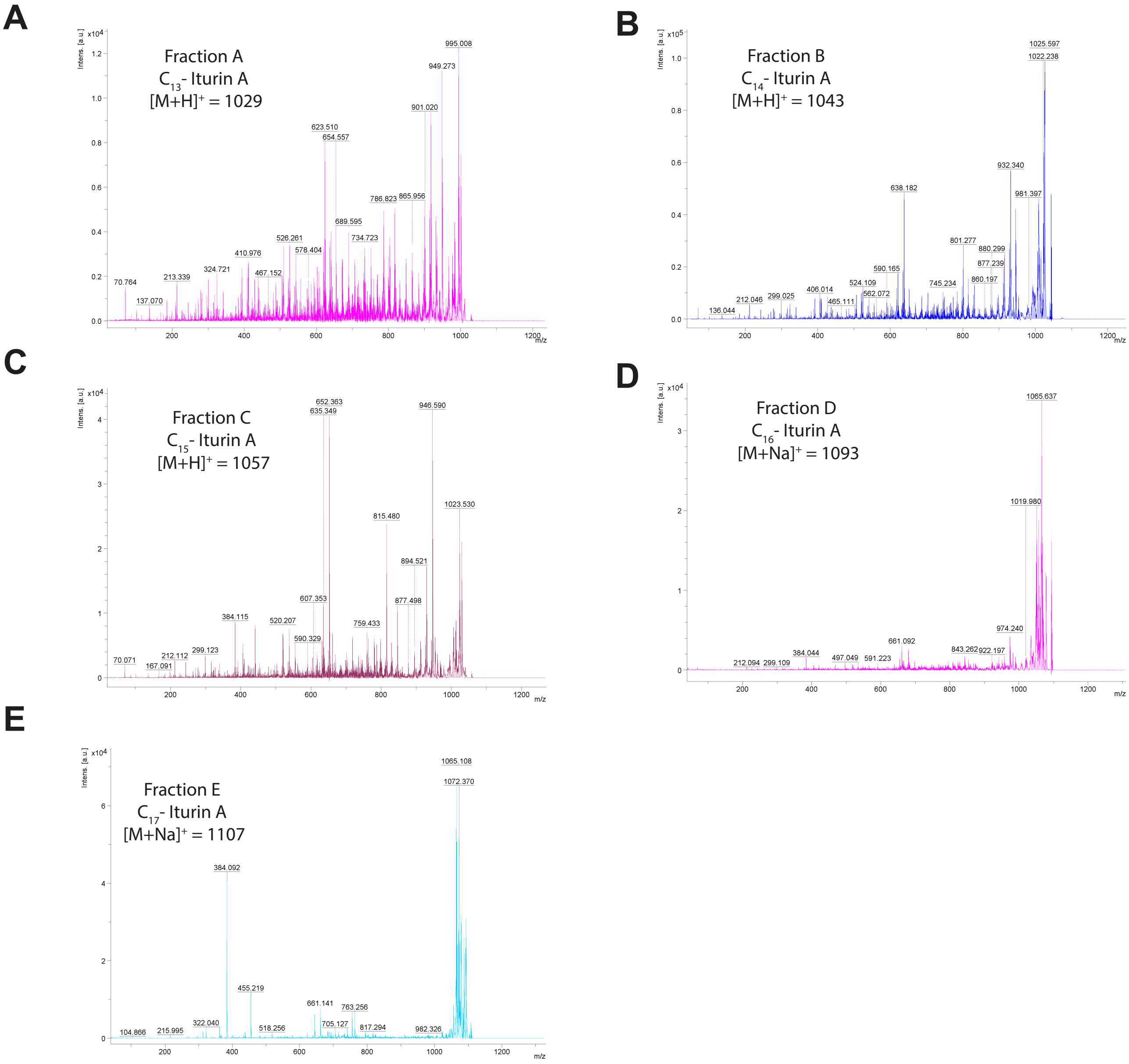

Figure S2. Mass spectra of purified iturin fractions (A-E). Double fragmentation was performed (MALDI-TOF MS/MS) to analyze the fragment ions of the selected precursor ions. m/z of the precursor ions is indicated in the upper part of each spectrum.

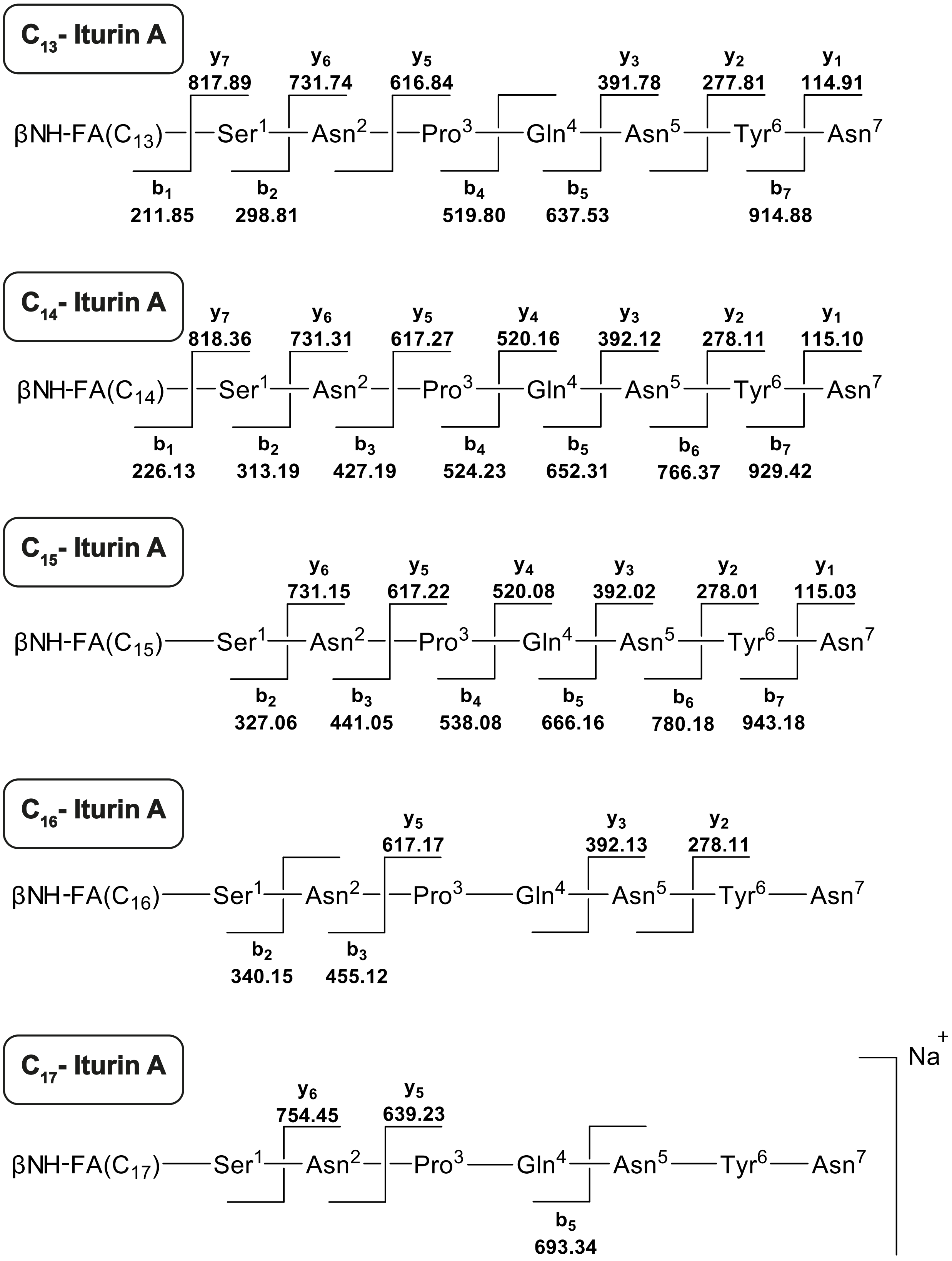

Figure S3. Proposed amino acid sequence corresponding to m/z peaks detected by MALDI-TOF MS/MS from iturin analogues fragmentation.

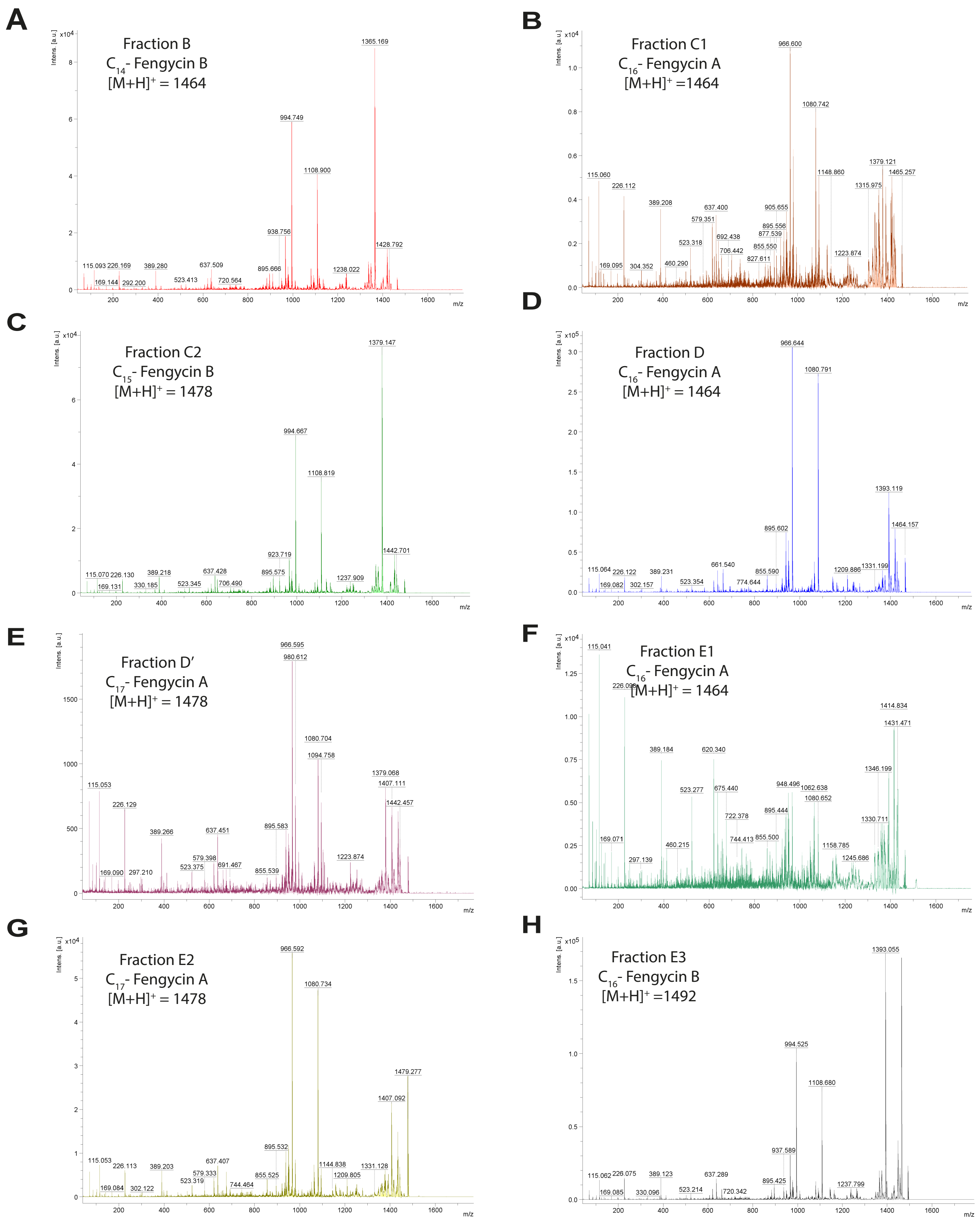

Figure S4. Mass spectra of purified fengycin fractions (A-H). Double fragmentation was performed (MALDI-TOF MS/MS) to analyze the fragment ions of the selected precursor ions. m/z of the precursor ions is indicated in the upper part of each spectrum.

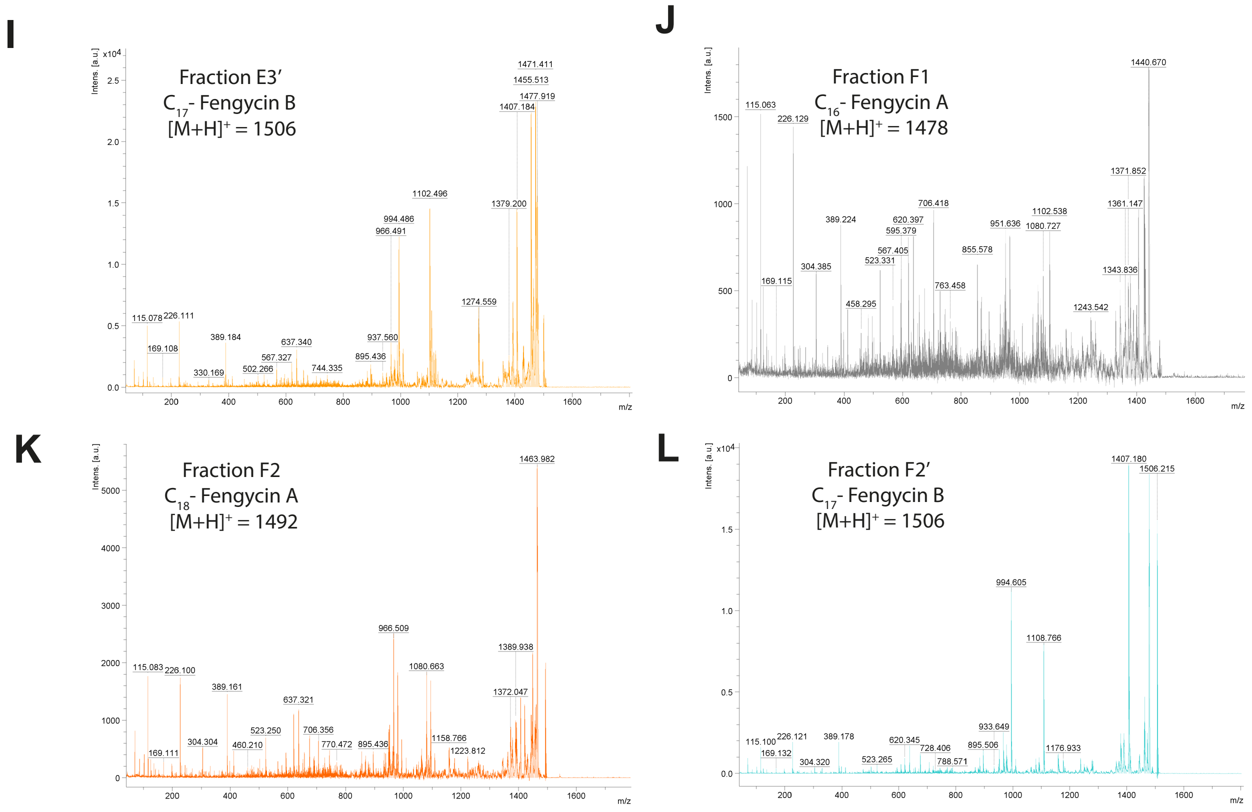

Figure S4. Mass spectra of purified fengycin fractions (I-L). Double fragmentation was performed (MALDI-TOF MS/MS) to analyze the fragment ions of the selected precursor ions. m/z of the precursor ions is indicated in the upper part of each spectrum.

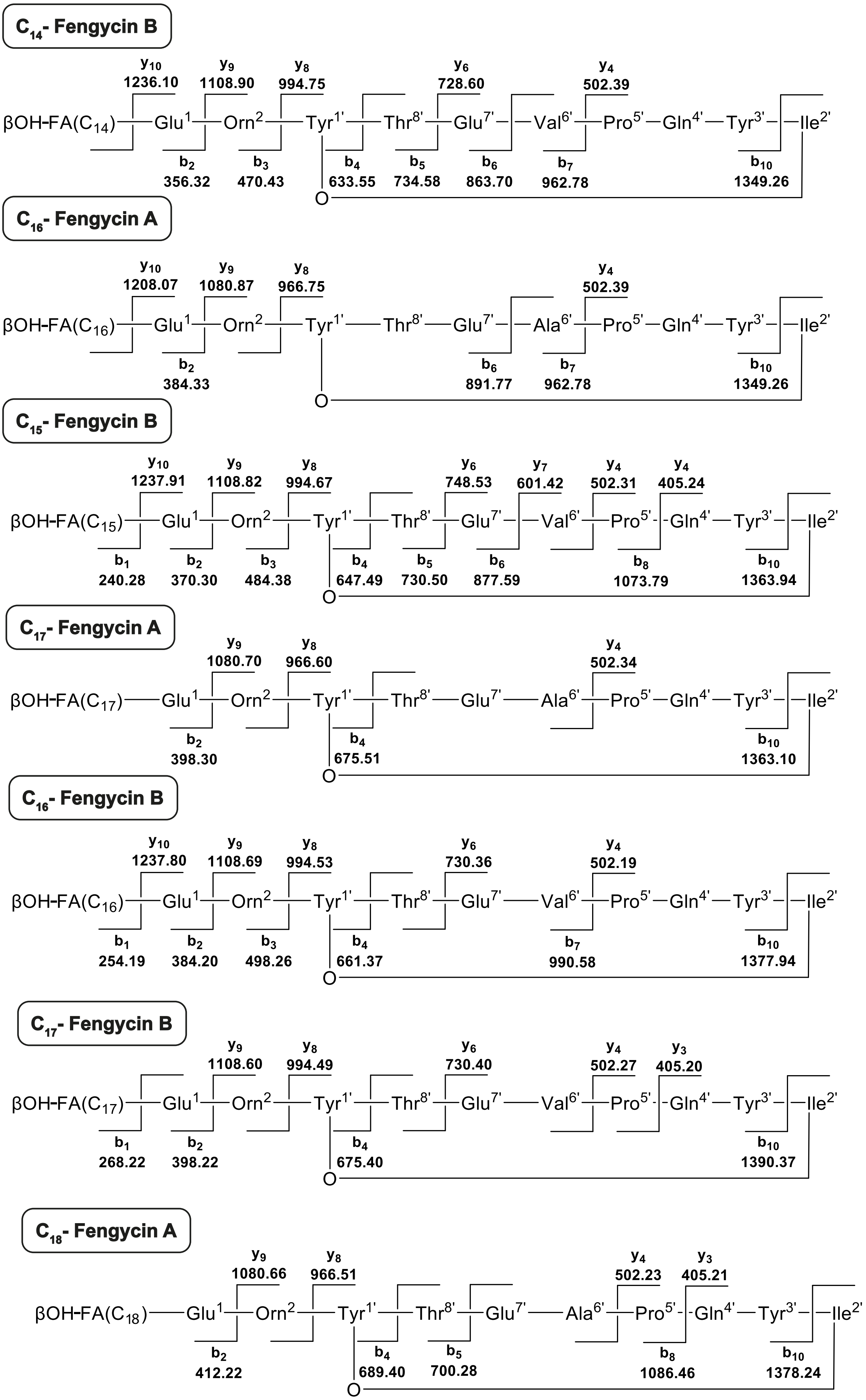

Figure S5. Proposed amino acid sequence corresponding to m/z peaks detected by MALDI-TOF MS/MS from fengycin analogues fragmentation.

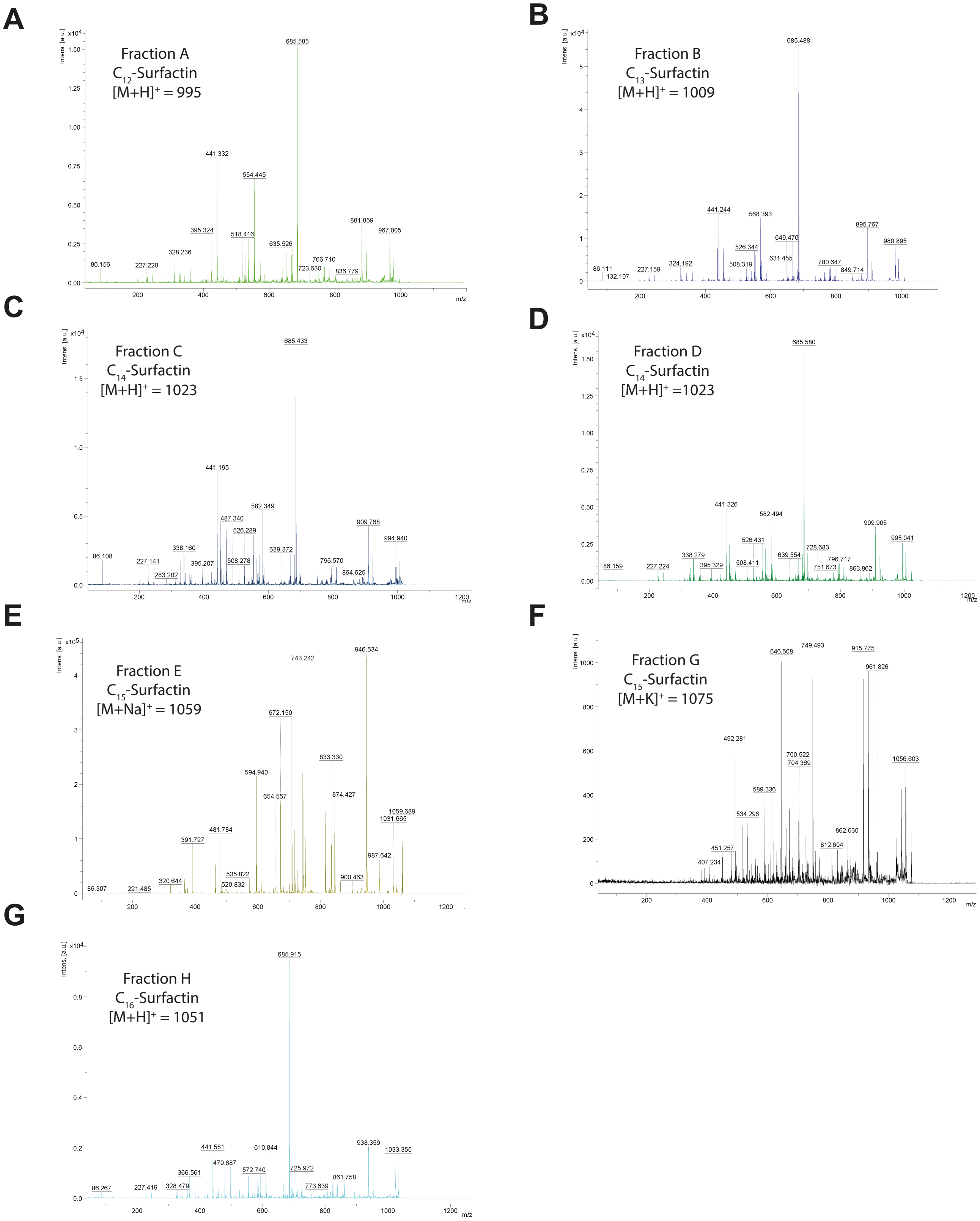

Figure S6. Mass spectra of purified surfactin fractions (A-G). Double fragmentation was performed (MALDI-TOF MS/MS) to analyze the fragment ions of the selected precursor ions. m/z of the precursor ions is indicated in the upper part of each spectrum.

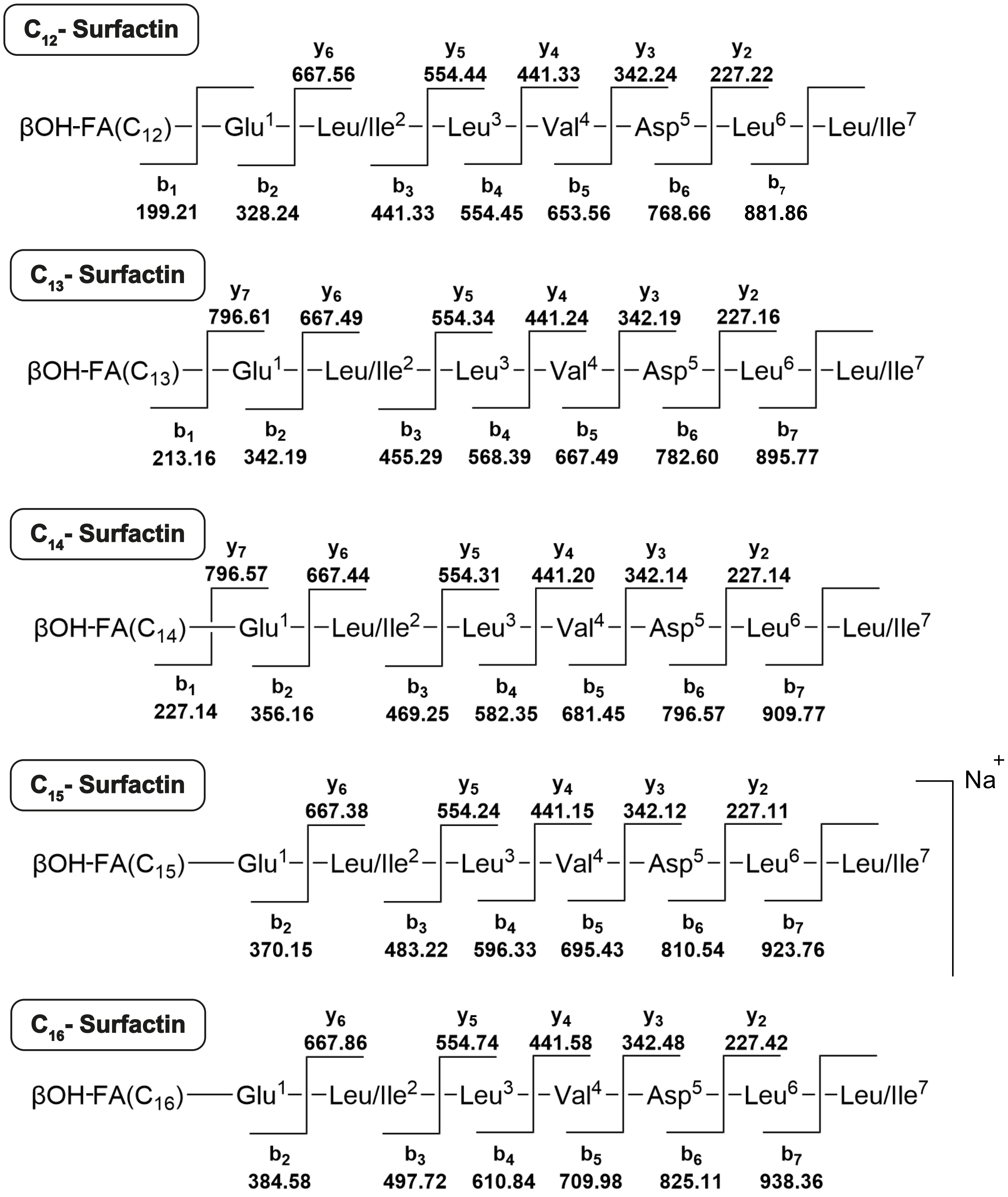

Figure S7. Proposed amino acid sequence corresponding to m/z peaks detected by MALDI-TOF MS/MS from surfactin analogues fragmentation.

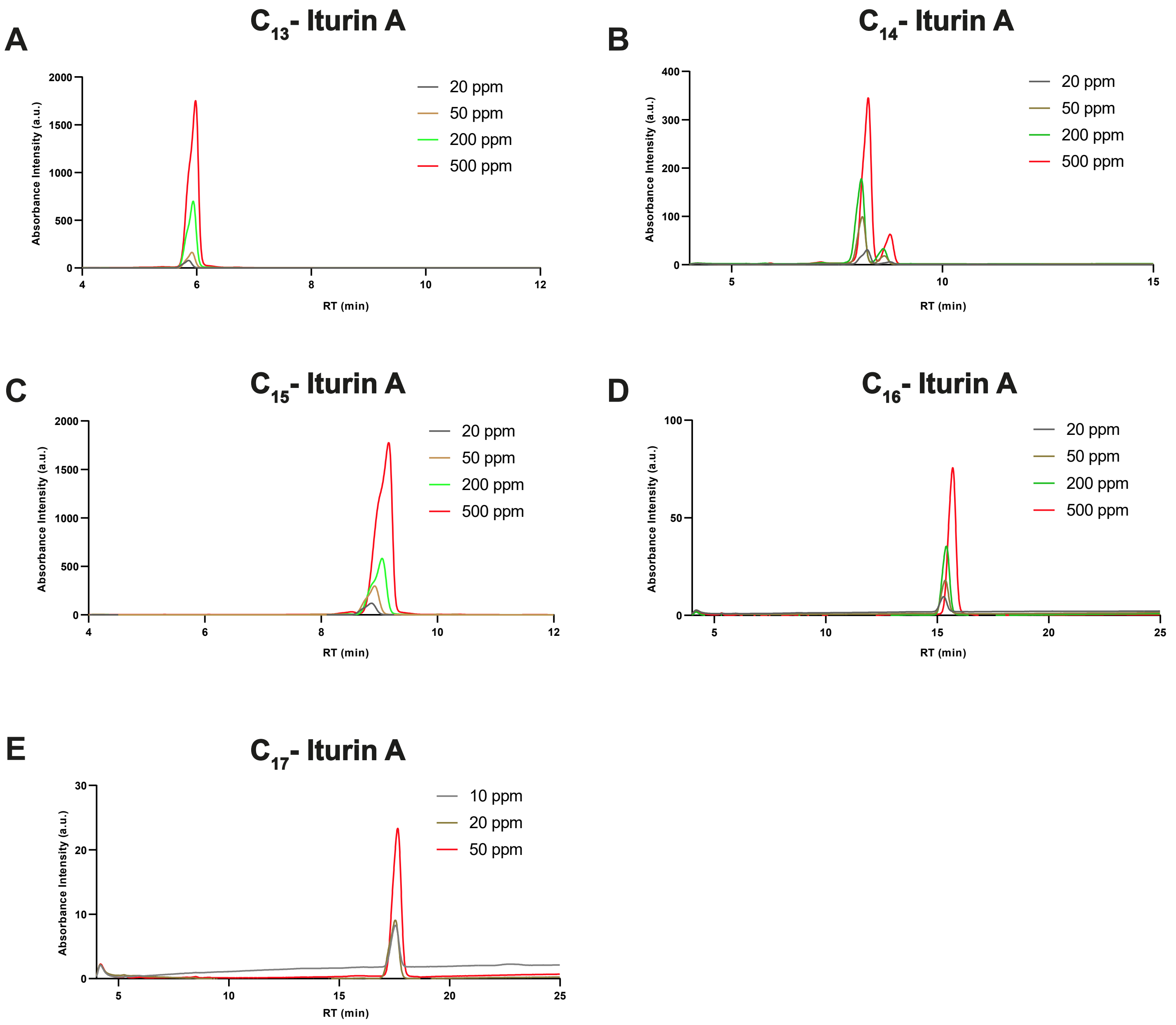

Figure S8. RP-HPLC chromatograms corresponding to calibration of iturin A analogues (A-E) purified from *B. velezensis* UMAF6639.

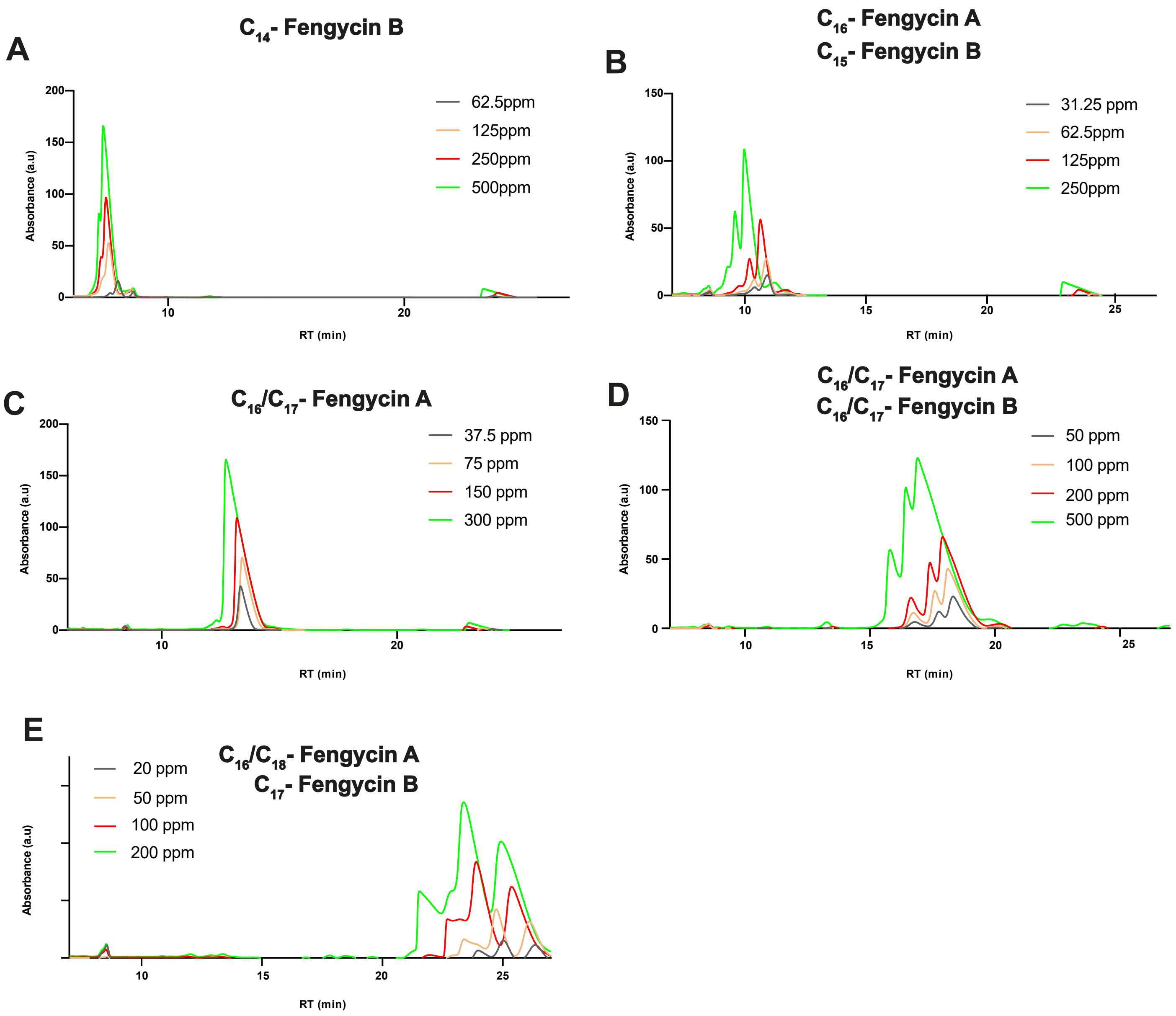

Figure S9. RP-HPLC chromatograms corresponding to calibration of fengycin analogues (A-E) purified from *B. velezensis* UMAF6639. Previously purified fractions were mixed according to their composition detected by tandem mass spectrometry.

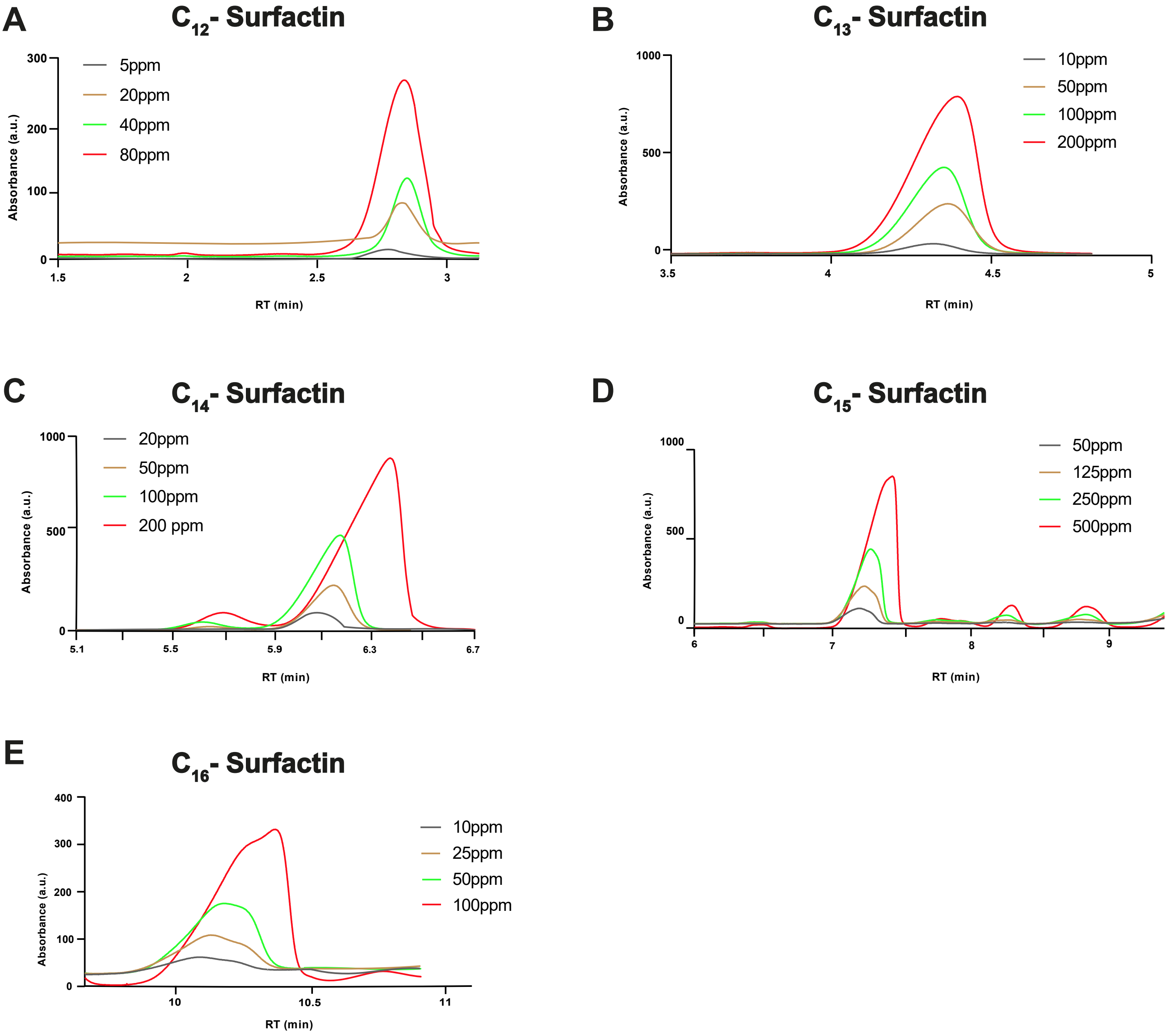

Figure S10. RP-HPLC chromatograms corresponding to calibration of surfactin analogues (A-E) purified from *B. velezensis* UMAF6639. Previously purified fractions were mixed according to their composition detected by tandem mass spectrometry.

Supplementary Tables

Table S1. Primers used to quantify the expression levels of lipopeptide genes by RT-qPCR.

Gene	Name	Sequence
rpsJ 30S ribosomal protein S10	rpsJ-Fw	5’-TCTGGTCCGATTCCGTTGCC-3’
	rpsJ-Rv	5’-CAGTTTGTGGTGTTGGGTTCA-3’
ituD First gene of the iturin biosynthetic operon	ituD-Fw	5’-GATGCGATCTCCTTGGATGT-3’
	ituD-Rv	5’-GCGCTGAATATTCGCCTAAG-3’
fenA First gene of the fengycin biosynthetic operon	fenA-Fw	5’-TGCGCTATATGCTCGATGAC-3’
	fenA-Rv	5’-AATCGCTATCCGCTTCTTCA-3’
srfAA First gene of the surfactin biosynthetic operon	srfAA-Fw	5’-AAGGAAACATCGTCACACAT-3’
	srfAA-Rv	5’-TTTAACAGCGAACCGAACAT-3’

Table S2. Linear regression equations corresponding to the calibration of each lipopeptide analog. In fractions containing more than one peak, total area was used for calculations.

Lipopeptide	Analog	Linear regression equation	R^2
**ITURIN**	C_{13}-Itu	Y = 40.120*X - 69.000	0.9978
	C_{14}-Itu	Y = 26.150*X - 325.200	0.9786
	C_{15}-Itu	Y = 162.500*X – 3463	0.9634
	C_{16}-Itu	Y = 9.344*X - 42.940	0.9955
	C_{17}-Itu	Y = 10.980*X + 75.560	0.9940
**FENGYCIN**	C_{14}-FengB	Y = 4.463*X + 7.582	0.9939
	C_{16}-FengA<br>C_{15}-FengB	Y = 5.036*X - 2.386	0.9954
	C_{16}/C_{17}- FengA	Y = 27.820*X - 73.700	0.9990
	C_{16}/C_{17}- FengA<br>C_{16}/C_{17}- FengB	Y = 24.090*X + 241.700	0.9997
	C_{17}/C_{18}- FengA<br>C_{17}- FengB	Y = 31.250*X - 149.100	0.9995
**SURFACTIN**	C_{12}-Surf	Y = 0.241*X + 0.401	0.9985
	C_{13}-Surf	Y = 0.931*X - 6.367	0.9952
	C_{14}-Surf	Y = 0.581*X - 7.482	0.9956
	C_{15}-Surf	Y = 2.255*X + 23.760	0.9982
	C_{16}-Surf	Y = 0.796*X - 3.686	0.9898

Table S3. Calculated physiological concentrations of the different lipopeptide analogues produced by *B. velezensis* UMAF6639.

	Lipopeptide fraction	Analog	Concentration (μM)
**ITURIN A**	Iturin Mix	-	7.35
	Fraction A	C_{13}-Itu	1.27
	Fraction B	C_{14}-Itu	2.39
	Fraction C	C_{15}-Itu	0.76
	Fraction D	C_{16}-Itu	1.96
	Fraction E	C_{17}-Itu	0.93
**FENGYCIN**	Fengycin Mix	-	25.60
	Fraction B	C_{14}-FengB	3.96
	Fraction C	C_{16}-FengA<br>C_{15}-FengB	5.44
	Fraction D	C_{16}/C_{17}- FengA	2.50
	Fraction E	C_{16}/C_{17}- FengA<br>C_{16}/C_{17}- FengB	11.21
	Fraction F	C_{17}/C_{18}- FengA<br>C_{17}- FengB	2.22
**SURFACTIN**	Surfactin Mix	-	65.83
	Fraction A	C_{12}-Surf	3.67
	Fraction B	C_{13}-Surf	16.93
	Fraction C	C_{14}-Surf	20.49
	Fraction D	C_{14}-Surf	
	Fraction E	C_{15}-Surf	21.85
	Fraction F	C_{15}-Surf	
	Fraction G	C_{15}-Surf	
	Fraction H	C_{16}-Surf	4.11

Table S4. Structure of the fatty acid side chains corresponding to iturin A homologues. (Adapted from [37]).

Analog	Homologues
**C_{13}-Iturin A**	CH_3-CH_2-
**C_{14}-Iturin A**	CH_3-CH_2-CH_2-
**C_{15}-Iturin A**	CH_3-CH_2-CH-<br>| CH_3
	$(CH_3)_2$-CH-CH_2-
	CH_3-CH_2-CH_2-CH_2-
**C_{16}-Iturin A**	$(CH_3)_2$-CH-CH-
	$(CH_3)_2$-CH-CH_2-CH_2-
**C_{17}-Iturin A**	CH_3-CH_2-CH_2-CH_2-CH_2-
